# The Lichen Genus *Sticta* (Lobariaceae, Peltigerales) in East African Montane Ecosystems

**DOI:** 10.3390/jof9020246

**Published:** 2023-02-12

**Authors:** Ulla Kaasalainen, Paul M. Kirika, Neduvoto P. Mollel, Andreas Hemp, Jouko Rikkinen

**Affiliations:** 1 Finnish Museum of Natural History, University of Helsinki, P.O. Box 7, 00014 Helsinki, Finland; 2 Department of Geobiology, University of Göttingen, Goldschmidtstraße 3, 37077 Göttingen, Germany; 3 National Museums of Kenya, East African Herbarium, Museum Hill Road, P.O. Box 45166, Nairobi 00100, Kenya; 4 National Herbarium, Tropical Pesticides Research Institute, P.O. Box 3024, Arusha 23201, Tanzania; 5 Department of Plant Systematics, University of Bayreuth, Universitätsstraße 30, 95440 Bayreuth, Germany; 6 Organismal and Evolutionary Biology Research Programme, Faculty of Biological and Environmental Sciences, University of Helsinki, P.O. Box 65, 00014 Helsinki, Finland

**Keywords:** Mt. Kilimanjaro, Taita Hills, Mt. Kasigau, Eastern Arc, Mt. Elgon, Eastern Afromontane biodiversity hotspot, nuITS, lichenized fungi, Ascomycota, molecular phylogeny

## Abstract

The lichen flora of Africa is still poorly known. In many parts of the tropics, recent studies utilizing DNA methods have revealed extraordinary diversity among various groups of lichenized fungi, including the genus *Sticta*. In this study, East African *Sticta* species and their ecology are reviewed using the genetic barcoding marker nuITS and morphological characters. The studied regions represent montane areas in Kenya and Tanzania, including the Taita Hills and Mt. Kilimanjaro, which belong to the Eastern Afromontane biodiversity hotspot. Altogether 14 *Sticta* species are confirmed from the study region, including the previously reported *S. fuliginosa*, *S. sublimbata*, *S. tomentosa*, and *S. umbilicariiformis*. *Sticta andina*, *S. ciliata*, *S. duplolimbata*, *S. fuliginoides*, and *S. marginalis* are reported as new to Kenya and/or Tanzania. *Sticta afromontana*, *S. aspratilis*, *S. cellulosa*, *S. cyanocaperata*, and *S. munda*, are described as new to science. The abundance of new diversity detected and the number of taxa represented by only few specimens show that more comprehensive sampling of the region may be needed to reveal the true diversity of *Sticta* in East Africa. More generally, our results highlight the need for further taxonomic studies of lichenized fungi in the region.

## 1. Introduction

Tropical mountains, and especially their forests, are hot spots of biodiversity and endemism [1,2,3,4]. In East Africa, montane regions, such as the ancient Eastern Arc Mountains which range from southern Tanzania to Kenya, and the much younger volcanic mountains, such as Mt. Kilimanjaro in Tanzania and Mt. Elgon at the border of Kenya and Uganda, are surrounded by vast stretches of arid woodlands and savannas [5,6]. Still, especially the humid upper windward slopes of these mountains have provided refuge for the montane rainforests already for millions of years [6,7]. During this time, climatic induced fluctuations in the areal extent and isolation of moist montane forests have generated remarkably high levels of diversity and local endemism [1,3,6,8,9] in what is now known as the Eastern Afromontane biodiversity hotspot [10,11].

The lichen flora of Africa is poorly known and is still largely based on information collected during the 20th century [12]. So far, only few groups of parmelioid and cyanolichens have been studied in any detail, and especially the more recent application of DNA methods has revealed high levels of previously unknown diversity [13,14,15,16,17,18]. *Sticta* (Schreb.) Ach. (Lobariaceae, Peltigerales) is a genus of foliose macrolichens characterized by well-differentiated pores called cyphellae on the lower surface. Recent molecular studies from different parts of the globe have demonstrated that the genus is very rich in species, with altogether 500 or more species [19,20,21,22,23]. Many traditionally delimited *Sticta* species, such as *S. fuliginosa* and *S. weigelii*, have been shown to represent complexes of taxa with a somewhat similar gross morphology, often called morphodemes [19,20,21,22,23]. High species diversity has been found from tropical montane areas of the Neotropics, and Madagascar and nearby islands [19,24,25]. In their classical guide to East African macrolichens, Swinscow and Krog [12] listed ten *Sticta* species from an East African region encompassing Ethiopia, Kenya, Tanzania, and Uganda. Since then, only one additional species has been reported from this region [26]. These reported taxa include *S. ambavillaria* (Bory) Ach., *S. cyphellulata* (Müll. Arg.) Hue*, S. dichotoma* Delise*, S. fuliginosa* (Hoffm.) Ach.*, S. kunthii* Hook. f., *S. limbata* (Sm.) Ach.*, S. orbicularis* (Braun) Hue*, S. papyracea* Delise*, S. sublimbata* (Steiner) Swinscow and Krog*, S. tomentosa* (Swartz) Ach., and *S. weigelii* var. *weigelii* (Ach.) Vainio and *S. weigelii* var. *xanthotropa* (Krempelh.) Hue [12,26,27,28,29,30,31], of which the latter is now known as *S. xanthotropa* (Kremp.) D. J. Galloway [32]. *Sticta papyracea* has been treated as a synonym of *S. variabilis* Ach. [33], and the reports of *Sticta limbata* from many parts of the world, including East Africa, probably represent *S. umbilicariiformis* Hochsc. Ex Flotow [20], a species originally described from Ethiopia. Additionally, *S. duplolimbata* (Hue) Vain. And *S. ciliata* Taylor have been reported from Rwanda [20].

In this study, we review *Sticta* species and their ecology in the montane regions of Kenya and Tanzania using the barcoding nuITS genetic marker and provide a wealth of new information on their diversity and ecology.

## 2. Materials and Methods

### 2.1. Study Locations and Sampling

Specimens were collected from four mountain regions in East Africa, including the dormant volcano Mt. Kilimanjaro in Tanzania, the Taita Hills, and Mt. Kasigau, which represent the northeastern end of the Eastern Arc Mountain Range in Kenya, and Mt. Elgon at the border of Kenya and Uganda. All the mountains are less than 400 km from the equator and separated from each other by wide semiarid plains with a tropical climate with two distinct rainy seasons.

The high Mt. Kilimanjaro (5895 m) supports a wide range of natural vegetation types from natural savanna to alpine *Helichrysum* heath, in addition to which human activity has produced a variety of additional habitat types. The sampling in the Kilimanjaro region was done in 2016–2017 along five replicate transects on the southern and southeastern slopes of the mountain. The 65 sampling plots represent the following 13 natural and disturbed ecosystems, with 5 replicate plots in each ecosystem type: natural savanna and maize fields (800–1100 masl), lower montane forests, traditional Chagga home gardens, commercial coffee farms, and grasslands (1100–2000 masl), montane *Ocotea* forest and selectively logged *Ocotea* forest (2100─2800 masl), upper montane *Podocarpus* forest and secondary forest dominated by *Erica excelsa* as a result of repeated forest fires (2800–3100 masl), subalpine *Erica trimera* forest and fire disturbed *E. trimera* forest/shrubbery (3500–4000 masl), and alpine *Helichrysum* heath (4000–4650 masl). For a more detailed description of the sampled environments on Mt. Kilimanjaro, see [13,34]. On each plot, lichen specimens were collected from a 5 × 20 m central plot and along two 50 m transects. Additionally, also a larger 20 × 50 m plot was sampled for branches that had dropped from the canopy.

The Taita Hills consists of three mountain massifs: Dabida, Mbololo, and Sagalla. While the potential natural vegetation on the moist upper slopes consists of evergreen *Ocotea* forest, long-lasting and intensive human influence has fragmented the indigenous forest into small and often heavily disturbed, isolated patches [35,36,37]. The sampling of lichens in the Taita Hills took place during several field trips mainly in 2009–2011, encompassing all the main remaining forest fragments: On the Dabida massif, fragments of indigenous montane forest mainly occur on the highest peaks and ridges, including Ngangao (120 ha), Chawia (86 ha), Yale (16 ha), Fururu (8 ha), Macha (3 ha), Mwachora (2 ha), Vuria (<1 ha), and Shomoto Hill (<0.2 ha) [36,38]. Mt. Sagalla in the southeast harbors a small indigenous forest patch of 2 ha surrounded by plantation forest, while Mbololo in the northeast has a relatively well-preserved 185 ha moist montane forest on top of a single ridge [39]. Additionally, few specimens were collected from Maktau Hill, an isolated peak with a small patch of dry woodland vegetation, lying west of the Dabida massif. All the collection localities in the Taita Hills were situated between 1300 masl (Maktau Hill) and 2208 masl (Vuria). For a more detailed description of the sampled forest fragments especially on the Dabida massif, see [13].

Mt. Kasigau is situated approximately 50 km southeast of the Taita Hills and, unlike the highly fragmented forests of the Taita Hills, the forest and woodland on Mt. Kasigau has remained relatively intact. The vegetation includes a transition from the *Acacia*-*Commiphora* bushland on the surrounding plains in 520 masl through a lower montane woodland to an evergreen forest, reaching the summit at 1641 masl [40,41]. On Mt. Kasigau, specimens were mainly collected in 2010, along four transects corresponding to the northern, eastern, southern, and western slopes of the mountain, as described in [42]. On Mt. Elgon, specimens were collected in 2016 from the ericaceous zone of the mountain.

Local species abundances in the Mt. Kilimanjaro sampling plots were defined based on species presence on the central plot and along the two transects, the value thus ranging from 0–3 for each sampling plot. Abundances in habitat types were defined as the sum of abundances in the five sample plots representing each habitat type. In the Taita Hills, abundance was defined as the number of different forest fragments from which a species was collected (0–11); abundance was not estimated for the individual forest fragments. On Mt. Kasigau, the abundance was defined as the number of elevational transects from which the species was collected (0–4).

### 2.2. Morphological Inspection

In sum, 373 *Sticta* specimens from Mt. Kilimanjaro, the Taita Hills, Mt. Kasigau, and Mt. Elgon (Table A1) were studied and identified based on morphological characters and the previous literature from the study region [12,26]. The morphological and anatomical characters were assessed using a Leica S8AP0 stereo and an Olympus BX51 compound light microscopes, the latter equipped with a Deltapix Invenio 12EIII camera. The description and naming of characters (branching, vegetative propagules, tomentum) follows Moncada et al. [43]. Spot reactions were checked from medulla with 10% KOH (K), sodium hypochlorite solution (liquid bleach; C), and 1,4-phenylenediamine in ethanol (Pd).

### 2.3. DNA Sequencing

The DNA extractions were made using the GeneJET Genomic DNA Purification Kit (Thermo Fisher Scientific, Waltham, MA, USA). For the extraction, a clean piece of lichen thallus (~0.1 cm^2^) was selected under a preparation microscope using a sterile needle or scalpel. Amplification and sequencing of the nuclear fungal internal transcribed spacer (ITS: ITS1-5.8S-ITS2) was performed as in [44], using primers ITS1 or ITS5, and ITS4 [45]. Sequencing was performed by Macrogen Europe (Amsterdam, the Netherlands) and LGC Genomics (Berlin, Germany). Sequences were edited with CodonCode Aligner [46]. The newly obtained ITS sequences were deposited in the NCBI GenBank database [47]. The specimen information, collection locations and the GenBank accession numbers are listed in Table A1.

### 2.4. Phylogenetic Analyses

The generated ITS sequence dataset was complemented with sequences downloaded from the GenBank [47]. The initial alignment of the ITS was done using MAFFT on the online server [48] and adjusted by hand using PhyDE v. 0.9771 [49]. Ambiguous regions were removed from the ITS alignment before the analysis of the complete dataset resulting an alignment of 346 sequences and 602 characters.

Bayesian analyses were performed using MrBayes v.3.2.7 [50,51] on CIPRES Science Gateway [52]. To allow possible deviating substitution models for the different regions the data sets were divided in a partition of three subsets (1: ITS1; 2: 5.8S; 3: ITS2). The best fitting nucleotide substitution models were selected by jModelTest [53] using AIC and BIC, and GTR + Γ was used for ITS1, SYM + Γ for 5.8S, and GTR + I + Γ for ITS2. Posterior probability distributions of trees were calculated using the Metropolis-coupled Markov chain Monte Carlo (MCMCMC) method and the search strategies suggested by Huelsenbeck et al. [54,55]. Three runs with four chains with 10 × 10^6^ generations each were run simultaneously. First, 25% of the trees were discarded (burnin), and the convergence of the runs confirmed with Tracer v. 1.7.1 [56] before the calculations for the 50% majority consensus tree and clade posterior probabilities (PP) were made. The trees were visualized using TreeGraph2 v2.15.0 [57].

To further analyze the phylogenetic relationships in the *Sticta umbilicariiformis*—*fuliginosa* clade, further analyses were run for selected specimens using more of the ITS region, following the same practices as in the first analysis. The alignment included 37 sequences and 495 characters with *S. duplolimbata* (KT281696), *S. andensis* (KC732547), and *S. pseudolimbata* (KC732564) as outgroup sequences. SYM + Γ was selected for ITS1, JC for 5.8S, and HKY + I for ITS2 as substitution models, and the analysis was run for 5 × 10^6^ generations. The sequence alignment files and the resulting tree files from the phylogenetic analyses are available in the Zenodo repository (https://zenodo.org/) with doi 10.5281/zenodo.7575780.

## 3. Results

Of the studied 373 *Sticta* specimens, a good quality ITS sequence was obtained from 233 specimens. ITS variant information for each sequenced lichen specimen is listed in Table A1.

### 3.1. Phylogenetic Analyses of the Specimens

The Bayesian analysis of the nuITS region of the genus *Sticta* revealed that several of the morphologically identified species included representatives of more than one phylogenetic lineage (Figure S1).

#### 3.1.1. Specimens with Soredia or Pustules

**Figure 1:**
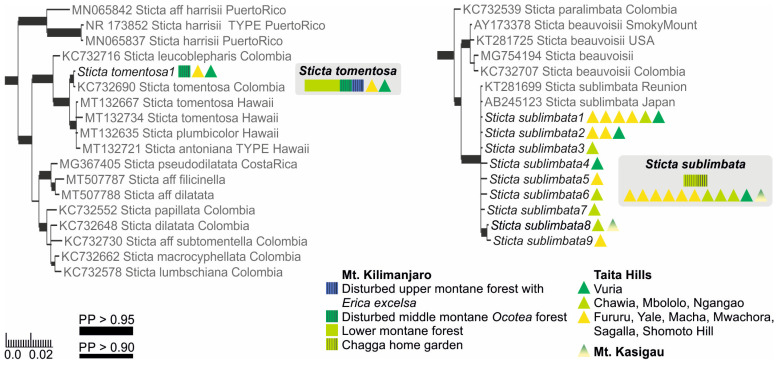
Clades with *Sticta tomentosa* and *S. sublimbata* of the Bayesian tree of the genus *Sticta* based on the nuITS marker region (Figure S1A,B). The colored polygons (rectangle, triangle) in the tree show the distribution of the ITS variants in the studied regions and ecosystem types: On Mt. Kilimanjaro, the different habitats are indicated by color and grid; the width of the rectangle indicate the number of sample plots in which the taxon was present in each ecosystem type (square = 1). In the Taita Hills, each triangle indicates presence in one forest fragment and on Mt. Kasigau in one sampling transect. The grey boxes show the total abundance and distribution of the species, also including the data from unsequenced specimens. Stronger support (PP > 0.9) for a clade is indicated with a thicker branch. The scale refers to nucleotide substitutions per site.

**Figure 2:**
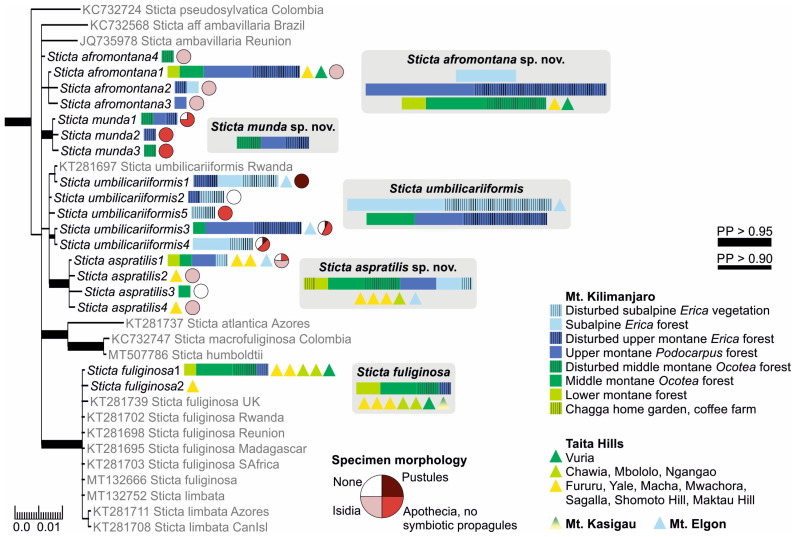
Phylogeny of the *Sticta umbilicariiformis—fuliginosa* group based on the nuITS region. The colored polygons (rectangle or triangle) in the tree show the distribution of the ITS variants in the studied regions and ecosystem types: On Mt. Kilimanjaro, the different habitats are indicated by color and grid; the width of the rectangle indicate the number of sample plots in which the taxon was present in each ecosystem type (square = 1). In the Taita Hills, each triangle indicates presence in one forest fragment and on Mt. Kasigau in one sampling transect. The grey boxes show the total abundance and distribution of the species, also including the data from unsequenced specimens. The proportions of different structures (pustules, isidia, or just apothecia) among the specimens with specific ITS variants are indicated with pie charts for the new species and *S. umbilicariiformis*. Stronger support (PP > 0.9) for a clade is indicated with a thicker branch. The scale refers to nucleotide substitutions per site.

Based on the previous literature from the region [12], the sorediate specimens were identified as either *S. sublimbata* or *S. limbata*. All specimens with soredia that were collected from the lower to middle montane forest zones formed a well-supported (PP = 1) clade together with *S. sublimbata* specimens from Réunion and Japan (Figure 1). However, the sorediate-pustular specimens from higher elevation habitats did not group together with *S. limbata*, but were closely related to a previously sequenced *S. umbilicariiformis* specimen from Rwanda (Figure 2).

#### 3.1.2. Fertile Specimens without Symbiotic Propagules

Three frequently fertile *Sticta* species that lack symbiotic propagules had previously been reported from the region, i.e., *S. ambavillaria*, *S. kunthii*, and *S. tomentosa* [12,26], and specimens resembling the descriptions of all these taxa were also present in our material. All specimens matching the description of *S. tomentosa* fell into a well-supported (PP = 1) clade, which mainly consists of *S. tomentosa* specimens from Colombia and Hawaii (Figure 1). All other specimens belonged to the *S. umbilicariiformis*—*fuliginosa* group (Figure 2). In the additional analysis, six specimens formed a well-supported (PP = 0.992) clade (*S. munda*); however, many morphologically similar specimens, i.e., fertile with pubescent or nodulous apothecial margins and with smooth, scrobiculate, foveolate to pitted upper surface, were mainly placed in the poorly resolved *S*. *umbilicariiformis* group, with some specimens in the well-supported (PP = 1) *S. aspratilis* clade.

#### 3.1.3. Specimens with Laminal Isidia (*Sticta fuliginosa* Morphodeme)

**Figure 3:**
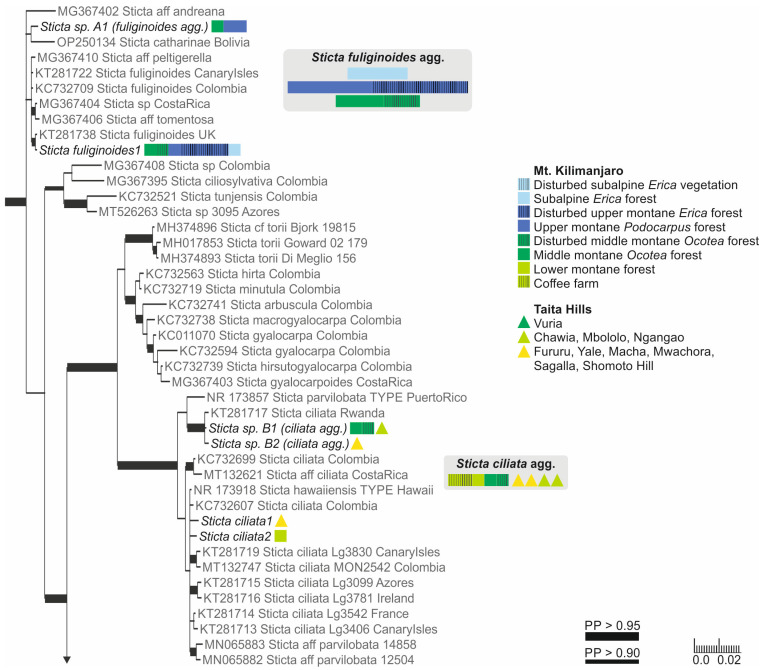
Clades with *Sticta fuliginoides*, *S. ciliata, Sticta* sp. A, and *Sticta* sp. B of the Bayesian tree of the genus *Sticta* based on the nuITS marker region (Figure S1C). The colored polygons (rectangle, triangle) in the tree show the distribution of the ITS variants in the studied regions and ecosystem types: On Mt. Kilimanjaro, the different habitats are indicated by color and grid; the width of the rectangle indicate the number of sample plots in which the taxon was present in each ecosystem type (square = 1). In the Taita Hills, each triangle indicates presence in one forest fragment. The grey boxes show the total abundance and distribution of the species, also including the data from unsequenced specimens. Stronger support (PP > 0.9) for a clade is indicated with a thicker branch. The scale refers to nucleotide substitutions per site.

Our specimens identified as *S. fuliginosa*, based on the previous literature from the region [12], fell into six different clades (Figure S1). These include three previously described species of the *Sticta fuliginosa* morphodeme, i.e., *S. ciliata*, *S. fuliginoides* (Figure 3), and *S. fuliginosa* (Figure 2). Additionally, four specimens (*Sticta* sp. B) representing two different ITS variants formed a well-supported (PP = 0.995) clade with one sequence obtained from a specimen from Rwanda identified as *S. ciliata*, forming a sister clade to *S. parvilobata*, a recently described species [23] from Puerto Rico (Figure 3). Additionally, three specimens (*Sticta* sp. A) were closely related to *S. catharinae*, another recently described species [22] from Bolivia (Figure 3). Nineteen specimens (*S. aspratilis*), mostly representing the *S. fuliginosa* morphodeme, but also including some fertile specimens without isidia, formed a well-supported (PP = 1) clade within the *S. umbilicariiformis*—*fuliginosa* group (Figure 2).

#### 3.1.4. Specimens of the *Sticta weigelii* Morphodeme

**Figure 4:**
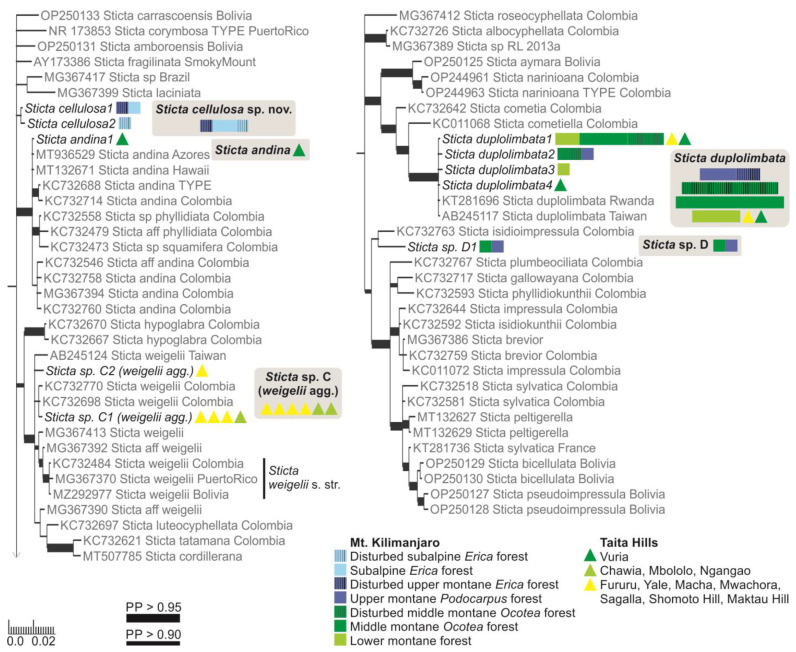
Clades with *Sticta cellulosa*, *S. andina*, *Sticta sp. C (weigelii agg.), S. duplolimbata*, and *Sticta* sp. D of the Bayesian tree of the genus *Sticta* based on the nuITS marker region (Figure S1D,E). The colored polygons (rectangle, triangle) in the tree show the distribution of the ITS variants in the studied regions and ecosystem types: On Mt. Kilimanjaro, the different habitats are indicated by color and grid; the width of the rectangle indicate the number of sample plots in which the taxon was present in each ecosystem type (square = 1). In the Taita Hills, each triangle indicates presence in one forest fragment. The grey boxes show the total abundance and distribution of the species, including also the data from unsequenced specimens. Stronger support (PP > 0.9) for a clade is indicated with a thicker branch. The scale refers to nucleotide substitutions per site.

The specimens belonging to the *Sticta weigelii* morphodeme, i.e., with cylindrical or flattened marginal isidia and often also with elongate lobes, were split into several different clades (Figure S1). The specimens with cylindrical isidia fell into a clade with specimens identified as *S. weigelii* from different parts of the world; however, the smaller clade consisting of specimens identified as *S. weigelii* s. str. [58] only includes GenBank sequences from the Neotropics (Figure 4).

**Figure 5:**
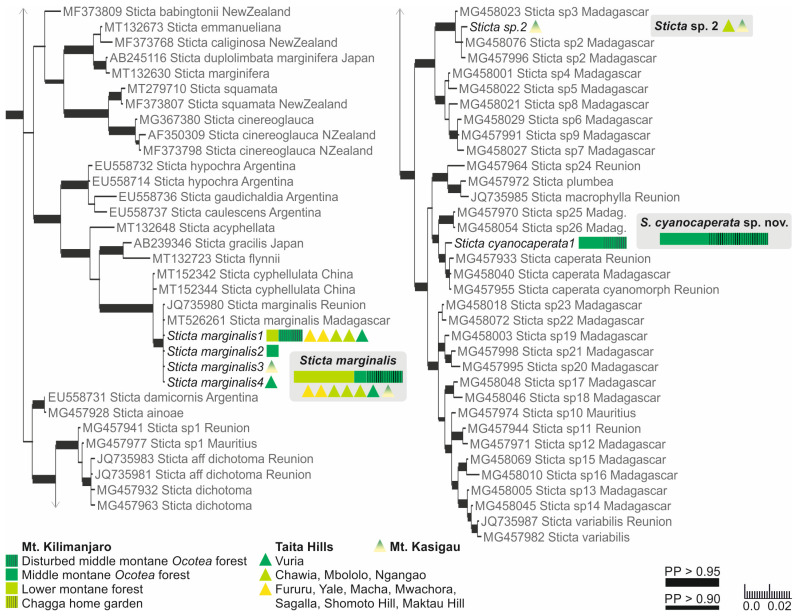
Clades with *Sticta marginalis*, *Sticta* sp. 2, and *Sticta cyanocaperata* in the Bayesian tree of the genus *Sticta* based on the nuITS marker region (Figure S1F). The colored polygons (rectangle, triangle) in the tree show the distribution of the ITS variants in the studied regions and ecosystem types: On Mt. Kilimanjaro, the different habitats are indicated by color and grid; the width of the rectangle indicate the number of sample plots in which the taxon was present in each ecosystem type (square = 1). In the Taita Hills, each triangle indicates presence in one forest fragment and on Mt. Kasigau in one sampling transect. The grey boxes show the total abundance and distribution of the species, also including the data from unsequenced specimens. Stronger support (PP > 0.9) for a clade is indicated with a thicker branch. The scale refers to nucleotide substitutions per site.

Specimens with at least some flattened isidia were divided into three different clades. The majority fell within the *S. umbilicariiformis*—*fuliginosa* group and formed a clade (PP = 0.665) closely related to *S. munda*, *S. umbilicariiformis*, and *S. aspratilis* (Figure 2). One specimen was placed into a well-supported (PP = 0.992) clade comprised mainly of *S. andina* specimens (Figure 4), and four specimens (*S. cyanocaperata*) grouped together with *S. caperata* from Réunion and Madagascar (Figure 5).

#### 3.1.5. Specimens with Marginal Isidia

Previously, two additional species with marginal isidia have been reported from the region, including *S. cyphellulata* and *S. orbicularis* [12]. Of the remaining marginally isidiate specimens, 18 clearly stipitate specimens formed a well-supported group with *S. marginalis* specimens from Réunion and Madagascar (Figure 5), and 22 formed a well-supported (PP = 1) group with *S. duplolimbata* specimens from other parts of the world (Figure 4). Additionally, specimens with cylindrical, mainly marginal isidia, but with an otherwise unique appearance (*S. cellulosa*), formed their own clade (PP = 0.868) among several recently described species mainly from the Neotropics (Figure 4). Additionally, two small and poorly developed specimens (*Sticta* sp. D) grouped (PP = 0.765) together with *S. isidioimpressula* but with a relatively long branch (Figure 4).

#### 3.1.6. *Sticta* with Green Algae

Previously, two *Sticta* species with a green algal photobiont have been reported from East Africa: *Sticta dichotoma* and *S. papyracea/variabilis* [12]. Only four such specimens were collected by us, all resembling the description of *S. papyracea*. However, in the phylogenetic analysis, the sequenced specimens did not group together with *S. variabilis* or *S. dichotoma*, but formed a clade (PP = 0.854) with some specimens from Madagascar (Figure 5), identified as “*Sticta* sp. 2” by Simon et al. [25].

### 3.2. Species of Sticta Identified

According to the results of the phylogenetic analysis, our *Sticta* specimens represent 19 distinct species (Table A1). These include nine previously established species, *S. andina*, *S. ciliata*, *S. duplolimbata*, *S. fuliginoides*, *S. fuliginosa*, *S. marginalis*, *S. sublimbata*, *S. tomentosa*, and *S. umbilicariiformis*; five newly described species, *S. afromontana*, *S. aspratilis*, *S. cellulosa*, *S. cyanocaperata*, *S. munda*, and five putative species, *Sticta* sp. A (*fuliginoides* agg.), *Sticta* sp. B (*ciliata* agg.), *Sticta* sp. C (*weigelii* agg.), *Sticta* sp. D, and *Sticta* sp. 2. Brief descriptions of the established species and full descriptions of the novel species are provided, including a summary of observations on their ecology and distribution. All the observed species are included in the key. *Sticta dichotoma* is included in the key based on previous published reports from the region [12].

#### 3.2.1. Key to *Sticta* Species in East Africa

**Figure 6:**
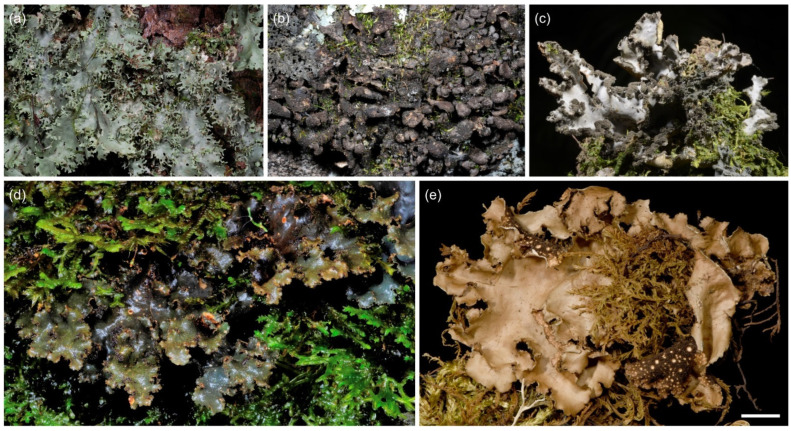
*Sticta* sp. 2, *Sticta* sp. B (*ciliata* agg.), *Sticta* sp. C (*weigelii* agg.), and *Sticta andina*. (**a**) *Sticta* sp. 2, the only green algal species collected by us, photographed in situ on Mt. Kasigau and showing the characteristic abundant and delicate marginal phyllidia (JR10K302). (**b**) *Sticta* sp. B, closely resembling *S. ciliata*, photographed in situ on Shomoto Hill and displaying the small, dark brown, rounded lobes and laminal isidia (JR10060). (**c**) *Sticta* sp. C photographed in situ in Sagalla forest with the characteristic congested heaps of cylindrical-coralloid isidia in the margins of the elongate lobes (JR16295). (**d**,**e**) *Sticta andina* (JR10117). (**d**) Photographed in situ on Vuria Mountain. (**e**) When dry, the species has a yellowish–brown upper surface and dark brown lower surface. Scale 0.5 cm in (**e**).

1a. Main photobiont green alga—2.
1b. Main photobiont cyanobacterium—3.
2a. Thallus thin and fragile, with marginal lobules—*Sticta* sp. 2 (Figure 6a)
2b. Thallus robust, without marginal lobules—*Sticta dichotoma*
3a. Thallus with marginal to submarginal soralia (may occasionally form coralloid, isidia-resembling structures) and/or pustules—4.
3b. Thallus without soredia or pustules—5.
4a. With true, mainly marginal soredia (found in sub/lower montane habitats, mainly <2000 masl)—***Sticta sublimbata***
4b. With pustules that may appear sorediate (found in upper montane and subalpine habitats, >3500 masl)—***Sticta umbilicariiformis***
5a. Thallus without symbiotic propagules, often fertile—6.
5b. Thallus with marginal or laminal isidia and/or phyllidia—9.
6a. Apothecial margin with abundant white hairs, not nodulous; lobe margins often with projecting tufts of hair/tomentum; cyphellae urceolate with a small pore; ascospores large (>40 × 8 µm in diam.)—***Sticta tomentosa***
6b. Apothecial margin slightly pubescent at most, most often nodulous; cyphellae not strongly urceolate with a small pore; ascospores shorter—7.
7a. Ascospores 3-septate; lobes usually <3 cm long and wide—***Sticta munda*** sp. nov.
7b. Ascospores 1-septate; lobes often larger—8.
8a. Ascospores 33–40 µm long—***Sticta umbilicariiformis***
8b. Ascospores usually shorter—***Sticta aspratilis*** sp. nov.
9a. Dark isidia marginal, submarginal, and on scrobiculate ridges present at least near the thallus margins of the brown thallus—***Sticta cellulosa*** sp. nov.
9b. Thallus without scrobiculate isidiate ridges—10.
10a. Isidia scattered over thallus lamina; without apothecia—11.
10b. Isidia predominantly on thallus margins (in fertile specimens often also on lamina)—14.
11a. Lobes elongate-obovate, fan-shaped, ascending from one attachment point with a funnel-like base; older thalli commonly with stalked lobules; lower side with pronounced, often clearly dome-like cyphellae (in middle montane to subalpine habitats, >2500 masl)—*Sticta fuliginoides* agg. (***Sticta fuliginoides*** and *Sticta* sp. A)
11b. Lobes rounded and/or palmate, without a funnel-shaped base, stalked lobules not present—12.
12a. Thallus small (up to 2 cm in diam.), lobes widely rounded (wider than long) and revolute (often complete lobes becoming convex); lower side marginally etomentose with widely different sized and often flat cyphellae (in lower and middle montane habitats, <2600 masl)—*Sticta ciliata agg*. (***Sticta ciliata*** and *Sticta* sp. B, Figure 6b)
12b. Thallus usually larger, lower side fully tomentose, cyphellae cupuliform to slightly urceolate—13.
13a. Lower side usually with abundant, arachnoid, moniliform secondary tomentum—***Sticta aspratilis*** sp. nov.
13b. Lower side usually without arachnoid, moniliform secondary tomentum—***Sticta fuliginosa***
14a. Lobes palmate, clearly stipitate and ascending from one attachment point; lower side largely etomentose, often with a yellow hue; marginal isidia developing into lobules especially in older thalli—***Sticta marginalis***
14b. Lobes not clearly stipitate, palmate, and ascending; lower side tomentose—15.
15a. Lobes elongate, with rounded apices, often with dark marginal cilia; cyphellae distinctly raised and urceolate with a small opening (dome-like)—***Sticta duplolimbata***
15b. Lobe margins without dark marginal cilia; cyphellae not distinctly dome-like—16.
16a. Isidia cylindrical to coralloid, in congested dark heaps, occasionally developing into stalked lobules—*Sticta* sp. C (*weigelii* agg.) (Figure 6c)
16b. Isidia flattened and extending horizontally from the margins, usually not in congested heaps—17.
17a. Lower surface color is usually cream to light brown with light to brown tomentum (or lower surface brown with white tomentum); upper surface grey to chocolate brown—18.
17b. Lower surface dark brown (at least centrally) with dark tomentum; upper surface color is usually fawn to yellow–brown (occasionally light grey or dark brown)—19.
18a. Thallus thick and large; with marginal flattened isidia; primary tomentum of agglutinated hyphae—***Sticta afromontana*** sp. nov.
18b. Rounded lobes with marginal and submarginal isidia and stalked lobules; lower surface at least centrally dark brown and glossy, tomentum white-light, only weakly agglutinated and hair-like, often entangled—*Sticta* sp. D
19a. K+ yellow; with moniliform secondary tomentum—***Sticta andina***
19b. K−; without moniliform secondary tomentum—***Sticta cyanocaperata*** sp. nov.

#### 3.2.2. *Sticta afromontana* Kaasalainen and Rikkinen sp. nov. (Figure 7)

Mycobank # MB847043

Species of *Sticta* lichenized with a cyanobacterium and characterized by robust thallus, flattened marginal isidia, and cream to mid–brown lower surface with moniliform secondary tomentum.

Type: **Tanzania**, Mt. Kilimanjaro, near the Maua Route, upper montane secondary forest with *Erica excelsa*, −3.1864° N 37.4403° E, 2820 masl, 11 March 2017, on a fallen branch, U. Kaasalainen UK170826e (H 9237169—holotype).

ITS barcoding marker accession (GenBank): OP999496 (holotype).

Description: *Thallus* rosetteform to irregular, 200–350 µm thick and up to 5 cm in diam., attached to substrate from the center of the lower side. *Lobes* robust, loosely adnate, elongate, palmate, up to 3 cm long and 2.5 cm wide, branching polytomous; margins crenate and crisped with abundant, mostly flattened isidia. *Upper surface* light grey to brown, smooth to slightly wavy to foveolate centrally, often with tufts of submarginal white hairs. *Upper cortex* paraplectenchymatous, 25–40 μm and 3(4) cell layers thick, the first layer(s) composed of smaller slightly flattened cells (~5 × 7 um), the others of larger (6–12 um) isodiametric cells. *Photobiont Nostoc. Photobiont layer* 45–85 μm thick, with *Nostoc* cells 5–6 μm in diam. *Medulla* 70–240 μm thick, with hyphae 2–4 µm wide. *Cilia* not present. *Isidia* abundantly present, mainly marginal but on fertile specimens also on lamina, grey to dark brown, glossy, coralloid, and mostly flattened and horizontal. *Lower surface* cream colored to (more rarely) brown, smooth or ridged, with abundant tomentum. *Lower cortex* paraplectenchymatous, 20–40 μm and 2–3 cell layers thick, with isodiametric cells 7–10 μm in diam*. Primary tomentum* usually with a brown and agglutinated stem, becoming white and squarrose towards the end. *Secondary tomentum* white, arachnoid, composed of moniliform assemblages. *Rhizines* infrequent, dark, slender. *Cyphellae* 45–90/cm^2^, cupuliform to slightly urceolate, with raised margins, pore up to 1.3 mm in diam., often with a darker ring surrounding the opening; cyphellar membrane white, 15–20 µm thick, composed of rounded, epapillose cells ~7 µm in diam. *Apothecia* infrequent and mainly found on specimens collected from optimal habitats; submarginal and laminal, sessile, up to 1.3 mm in diam. and 0.6 mm high (from the lower cortex of the lobe invagination); disc orange–brown; margin light brown with dark brown nodules. *Exciple margin* 95–115 μm wide. *Epithecium* 10–15 μm thick, orange–brown. *Hymenium* 100–120 μm thick. *Hypothecium* 55–70 µm thick, orange–brown. *Ascospores* fusiform, colorless, (1)3(5)-septate, 27–40 × 6–8 µm. *Pycnidia* not seen. *Chemistry*: K−, C−, Pd−.

**Figure 7:**
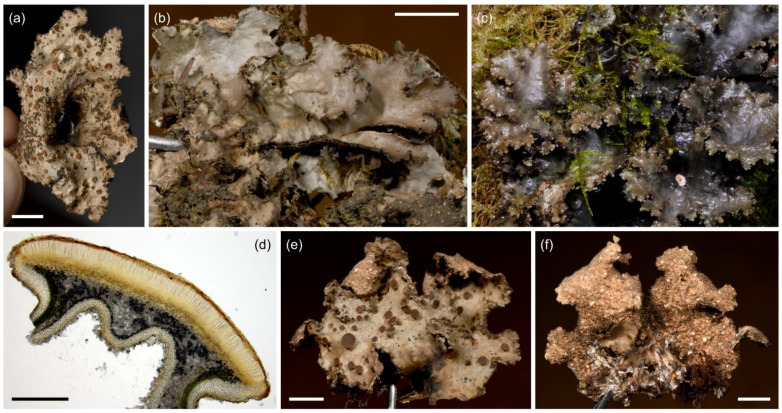
*Sticta afromontana*. (**a**) Type specimen UK170826e collected from the upper montane forest on Mt. Kilimanjaro. The specimen has laminal isidia that are not present in specimens without apothecia. (**b**) Specimen JR16366 illustrating typical habit with marginal isidia. (**c**) Specimen JR10112 photographed in situ on Vuria Mountain. (**d**) Cross section of an apothecium from the type specimen. (**e**,**f**) Thallus part of the same collection as the type imaged from the upper and lower side. Scales 0.5 cm in (**a**), (**b**), (**e**), and (**f**), 300 µm in (**d**).

Etymology: Sticta *afromontana* is one of the most common *Sticta* species in the studied afromontane region.

Ecology and distribution: *Sticta afromontana* is common, abundant and often fertile in the upper montane forest zone and present from lower montane forests to the ericaceous zone (1800–3510 masl) but has not been collected outside forest habitats. *Sticta afromontana* is particularly common on Mt. Kilimanjaro, where often the most abundant *Sticta* species, but also found in some Taita Hills forests. Epiphytic on tree trunks, branches, shrubs, and climbers. So far, only known from Tanzania and Kenya.

Selected specimens examined: **Tanzania**, Mt. Kilimanjaro, near Machame Route, lower montane forest, −3.1675° N 37.2363° E, 1920 masl, 15 March 2017, on a fallen branch, U. Kaasalainen UK170930c (H). Middle montane *Ocotea* forest, −3.0812° N 37.1444° E, 2260 masl, 9 March 2017, on fallen branch, U. Kaasalainen UK170792d (H). Upper montane *Podocarpus* forest, −3.1035° N 37.2604° E, 2850 masl, 9 March 2017, on mossy liana U. Kaasalainen UK170775b, U. Kaasalainen UK170779c. On fallen branch, U. Kaasalainen UK170781b (H). Near Marangu Route, upper montane *Podocarpus* forest, −3.1044° N 37.3046° E, 2800 masl, 13 March 2017, on tree trunk, U. Kaasalainen UK170886a (H). Near Maua Route, disturbed middle montane *Ocotea* forest, 2270 masl, on fallen branch, U. Kaasalainen UK171180a (H). Upper montane *Podocarpus* forest, −3.1936° N 37.4421° E, 2270 masl, 11 March 2017, on tree trunk, U. Kaasalainen UK170857a (H). On trunk of fallen tree, U. Kaasalainen UK170858g (H). Upper montane secondary forest with *Erica excelsa*, −3.1898° N 37.4390° E, 2880 masl, 11 March 2017, on a shrub branch, U. Kaasalainen UK170846e (H). −3.1864° N 37.4403° E, 2720 masl, 11 March 2017, on a fallen branch, U. Kaasalainen UK170806j (H). On a tree trunk, U. Kaasalainen UK170832c (H). Near the Mweka route, upper montane *Podocarpus* forest, −3.1659° N 37.3626° E, 2940 masl, 24 June 2017, on a fallen branch, U. Kaasalainen UK171526a (H). On a tree trunk, U. Kaasalainen UK171528b (H). Upper montane secondary forest with *Erica excelsa*, −3.1640° N 37.3675° E, 2990 masl, 25 June 2017, on a tree trunk, U. Kaasalainen UK171577f (H). Near the Umbwe route, middle montane *Ocotea* forest, −3.0824° N 37.1811° E, 2540 masl, 20 June 2017, on a tree trunk, U. Kaasalainen UK171490e (H). **Kenya**, Taita Hills, Vuria Mountain, −3.24° N 38.17° E, 2200 masl, J. Rikkinen JR10112 (EA), J. Rikkinen JR10121B (EA), J. Rikkinen and P. M. Kirika JR16366 (EA).

Notes: *Sticta afromontana* can be distinguished from other *Sticta* species in the region by its flattened marginal isidia and lack of K reaction (strong and immediate yellow in *S. andina*), presence of moniliform secondary hyphae (not present in *S. cyanocaperata*), and usually cream to light brown lower surface and primary tomentum (usually dark brown in *S. andina* and *S. cyanocaperata*). *Sticta xanthotropa*, previously reported from East Africa, has a thin and fragile thallus (robust in *S. afromontana)*, shorter ascospores (24–36 µm), and different substrate ecology (grows on rocks and soil) [32] than *S. afromontana*.

#### 3.2.3. *Sticta andina* B. Moncada, Lücking and Sérus. (Figure 6d,e)

A detailed description of *S. andina* is provided in [21]. Only one specimen of *S. andina* was identified from our material. It has a cyanobacterial main photobiont (*Nostoc*) and is characterized by flattened marginal isidia, yellowish–brown upper surface and dark brown lower surface with dark, short primary tomentum, and moniliform secondary tomentum. Chemistry: K+ yellow, C−, Pd−.

Morphologically, *S. andina* most resembles *S. cyanocaperata*; however, it can be easily identified based on the immediate, bright yellow K+ reaction in the medulla (*S. andina* is the only K+ species in our region). *Sticta cyanocaperata* also lacks the moniliform secondary tomentum present in *S. andina*.

Ecology and distribution: In other parts of the world (Columbia, Hawaii, Azores), *S. andina* has been reported to grow epiphytically in humid montane forests and in montane heathlands [21]. Our single specimen was collected from a moist montane forest on Vuria Mountain in the Taita Hills, and it shared an identical ITS sequence with a specimen previously collected from Hawaii (MT132671).

#### 3.2.4. *Sticta aspratilis* Kaasalainen and Rikkinen sp. nov. (Figure 8)

Mycobank # MB847044

Species of *Sticta* lichenized with a cyanobacterium and characterized by its large thallus, rough upper surface with laminal isidia, abundant moniliform secondary tomentum on the lower side, and short one-septate ascospores.

Type: **Kenya**, Mount Elgon National Park, ericaceous zone, 2016, on a tree trunk, J. Rikkinen and P. M. Kirika JR16107 (EA—holotype).

ITS barcoding marker accession (GenBank): OP999437 (holotype).

Description: *Thallus* rosetteform to irregular, attached to substrate from the center of the lower side. *Lobes* 130–250 µm thick, loosely adnate, rarely ascending, usually rounded palmate, usually 2–4 cm wide and 2–3 cm long, but sometimes more elongate and up to 7 cm long; branching polytomous, margins entire to sinuose, sometimes slightly crisped with isidia, occasionally revolute. *Upper surface* grey–brown or more rarely brown, usually at least slightly glossy, uneven, ridged (isidiate specimens) and/or foveolate, with occasional eroded patches surrounded by isidia. *Upper cortex* paraplectenchymatous, 25–60 μm and 3–6 cell layers thick, composed of tightly packed cells of ~7 µm in diam. *Photobiont Nostoc*. *Photobiont layer* 30–75 μm thick, with *Nostoc* cells 5–6 μm in diam. *Medulla* 35–145 μm thick, with hyphae 3–4 µm wide. *Cilia* not present. *Isidia* abundantly present, laminal, and sometimes also present on the lobe margins, brown or grey, darker than the upper surface, glossy, nodular to branched or coralloid. *Lower surface* cream to light brown, smooth to occasionally slightly uneven, abundantly tomentose. *Lower cortex* paraplectenchymatous, 20–40 μm and 3–4 cell layers thick, with cells 7–10 μm in diam*. Primary tomentum* white to brown, composed of agglutinated hyphae. *Secondary tomentum* abundant, pale, arachnoid, composed of moniliform assemblages. *Rhizines* sparse, in scattered groups, long, slender, concolorous with primary tomentum. *Cyphellae* 90–270/cm^2^, cupuliform to slightly urceolate, with a raised margin, very variable is size, pore up to 1(2.4) mm in diam.; cyphellar membrane white, ~20 µm thick, with rounded, epapillose cells ~8 µm in diam. *Apothecia* occasional, only seen on specimens without isidia; submarginal and laminal, up to 2.7 mm wide; margin beige to brown with darker brown stripes or nodules, sometimes slightly pubescent or tomentose; disc red–brown. *Exciple margin* 120–150 μm wide. *Epithecium* 10–20 µm thick, orange–brown. *Hymenium* 90–140 µm thick. *Hypothecium* 50−75 µm thick, orange–brown. *Ascospores* fusiform, 1-septate, colorless, (21)26–33(35) × 5.5–8 µm in diam. *Pycnidia* not seen. *Chemistry*: K−, C−, Pd−.

**Figure 8:**
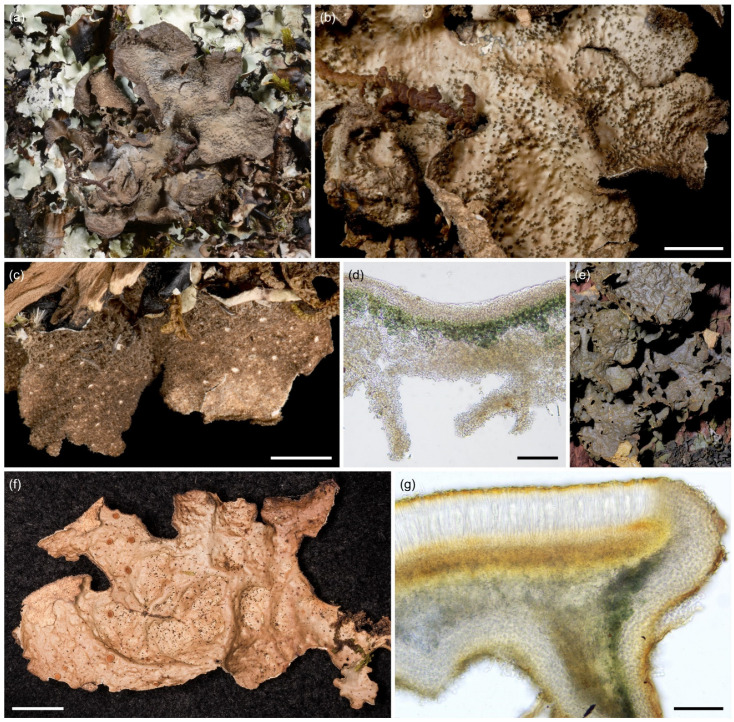
*Sticta aspratilis*. (**a**–**d**) Type specimen JR16107. (**a**) Photographed in situ in the ericaceous zone on Mt. Elgon. (**b**) The uneven and isidiate upper surface. (**c**) Abundantly tomentose lower surface. (**d**) Thallus cross section with a cyphella, showing the cyphellar membrane with rounded cells without papillae. (**e**) Specimen JR10155 photographed in situ on Yale, with gray and distinctly reticulate upper surface. (**f**,**g**) Fertile specimen UK171478a. (**f**) Foveolate upper surface with apothecia. (**g**) Cross section of an apothecium. Scales 0.5 cm in (**b**) and (**c**), 100 µm in (**d**) and (**g**), 1 cm in (**f**).

Etymology: The specific epithet refers to the characteristic uneven roughness of the upper thallus surface.

Ecology and distribution: *Sticta aspratilis* is relatively common, but not very abundant in any habitat type. It has a wide elevational range on Mt. Kilimanjaro, the Taita Hills, and Mt. Elgon, extending from lower montane forests to the subalpine zone (1450–3720 masl). In addition to primary forests, it has also been collected from disturbed habitats. Epiphytic, mainly on canopy branches, but also on tree trunks at more open sites, occasionally also on soil in the subalpine zone. So far, *S. aspratilis* is only known from Kenya and Tanzania.

Selected specimens examined: **Tanzania**, Mt. Kilimanjaro, coffee farm in Maua, −3.1630° N 37.2813° E, 1660 masl, 23 May 2017, on coffee bush, U. Kaasalainen UK170975f (H). Near Machame Route, upper montane *Podocarpus* forest, −3.1035° N 37.2604° E, 2970 masl, 9 March 2017, on fallen branch, U. Kaasalainen UK170781c (H). Near Marangu Route, upper montane *Podocarpus* forest, −3.1044° N 37.3046° E, 2800 masl, 13 March 2017, on tree trunk, U. Kaasalainen UK170892c (H). On fallen branch, U. Kaasalainen UK170896b (H). Near Mweka Route, middle montane *Ocotea* forest near Mweka Route, −3.1722° N 37.3583° E, 2850 masl, 24 June 2017, on a fallen branch, U. Kaasalainen UK171515b (H). Upper montane *Podocarpus* forest, −3.1616° N 37.3632° E, 2970 masl, 26 June 2017, on a fallen tree, U. Kaasalainen UK171587j (H). Disturbed subalpine *Erica* vegetation, −3.1339° N 37.3702° E, 3720 masl, 25 June 2017, on shrub base, U. Kaasalainen UK171562c (H). Near Umbwe Route, middle montane *Ocotea* forest, −3.0819° N 37.1819° E, 2650 masl, 20 June 2017, on fallen branch, U. Kaasalainen UK171478a (H). **Kenya**, Taita Hills, field edge near the Fururu Forest, −3.25° N 38.20° E, 1650 masl, 20 January 2011, on tree trunk, U. Kaasalainen UK110551f (EA), UK110551g (EA). Shomoto Hill, −3.395° N 38.360° E, 1500 masl, 2010, epiphytic, J. Rikkinen JR10044A (EA), JR10057 (EA). Yale, −03.24° N 38.20° E, 1850 masl, 2010, epiphytic, J. Rikkinen JR10155A (EA), JR10155B (EA), JR10155C (EA), JR10171 (EA).

Notes: *Sticta aspratilis* most closely resembles *S. fuliginosa* which, however, usually lacks the moniliform secondary tomentum that characteristically covers the lower surface of *S. aspratilis*. Fertile specimens may resemble *S. kunthii*, previously reported from East Africa, and fertile specimens of *S. umbilicariiformis*. However, the upper surface of *S. kunthii* is marbled with maculae and papillate [32], while *S. umbilicariiformis* usually has longer ascospores (33–40 µm) than *S. aspratilis*.

#### 3.2.5. *Sticta cellulosa* Kaasalainen sp. nov. (Figure 9)

Mycobank # MB847045

Species of *Sticta* lichenized with a cyanobacterium and characterized by its thick, brown lobes and tomentum, and the scrobiculate, isidiate ridges on the upper surface present at least near the margins.

Type: **Tanzania**, Mt. Kilimanjaro, near Umbwe Route, subalpine *Erica trimera* forest, −3.1114° N 37.3183° E, 3500 masl, 18 June 2017, on fallen branch, U. Kaasalainen UK171406e (H9237170—holotype).

ITS barcoding marker accession (GenBank): OP999548 (holotype).

Description: *Thallus* irregular, 150–300 µm thick, attached to substrate from the lower side of thallus. *Lobes* loosely adnate to ascending, elongate or rarely palmate, up to 3 cm wide and 4 cm long, branching polytomous, margins entire to sinuose, usually abundantly isidiate. *Upper surface* middle to dark brown or occasionally lighter yellow–brown, moderately glossy, smooth to foveolate centrally, with a scrobiculate pattern of isidiate ridges and hollows, at least submarginally, but occasionally spreading over a large part of the upper surface. *Upper cortex* paraplectenchymatous, 30–50 μm and 3–5 cell layers thick, cells up to 15 µm in diam., the first layer of cells often smaller and brownish in color. *Photobiont Nostoc*. *Photobiont layer* 20–70 μm thick, with *Nostoc* cells approximately 9 μm in diam. *Medulla* 50–180 μm thick, with hyphae 2–4 µm wide. *Cilia* not present. *Isidia* abundantly present and congested, marginal, submarginal, and on the laminal scrobiculate ridges, dark brown to almost black, glossy, cylindrical to coralloid, occasionally present also on the lower surface where grey. *Lower surface* dark brown, occasionally lighter towards margins, smooth, thickly and densely tomentose throughout. *Lower cortex* paraplectenchymatous, brown, 25–70 μm and 3–5 cell layers thick, with cells 6–14(20) μm in diam*. Primary tomentum* dark brown to almost black, composed of agglutinated hyphae. *Secondary tomentum* arachnoid, pale, composed of moniliform assemblages. *Rhizines* often present in small, scattered groups, clearly longer than tomentum, fasciculate, squarrose, dark brown and often with white tips. *Cyphellae* 13–50/cm^2^, cupuliform with a round pore and raised margins, larger may be more irregular and slightly urceolate, pore size very variable, up to 2.3 mm in diam.; cyphellar membrane cream-colored to slightly brown or yellow, 15–25 µm thick, composed of rounded, epapillose cells 5–12 µm in diam. *Apothecia* or *pycnidia* not seen. *Chemistry*: K−, C−, Pd−.

**Figure 9:**
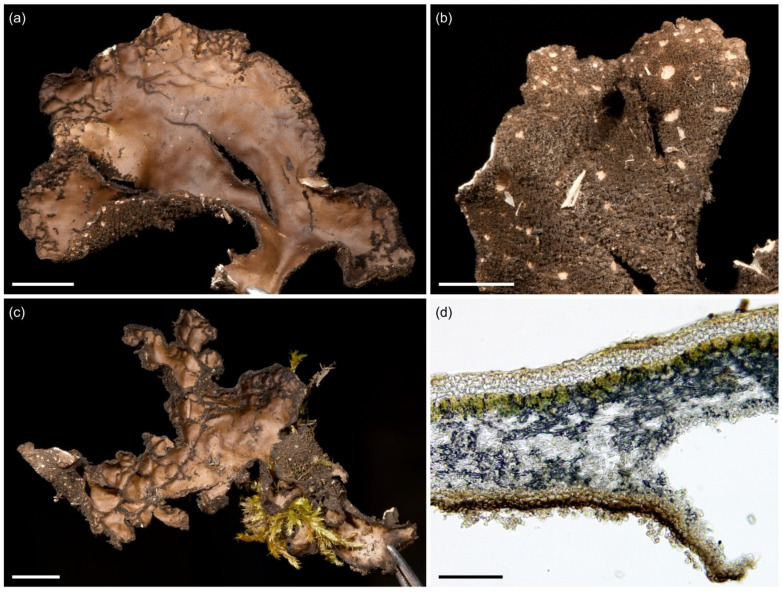
*Sticta cellulosa*. (**a**,**b**) Type specimen UK171406e imaged from the upper and lower side. (**c**) Thallus part of the same collection as type specimen with abundant characteristic isidiate ridges. (**d**) Thallus cross section with a cyphella on the right showing the epapillate cells of the cyphellar membrane, and the moniliform assemblages which form secondary tomentum on the lower surface (UK171340t). Scales 0.5 cm in (**a**–**c**), 100 µm in (**d**).

Etymology: The specific epithet refers to the characteristic reticulate pattern of the upper thallus surface.

Ecology and distribution: *Sticta cellulosa* seems to be rare and is found mainly in the subalpine zone on Mt. Kilimanjaro (2990–3520 masl). It occurs in primary and secondary (fire disturbed) *Erica trimera* forests and in the secondary upper montane forest with *Erica excelsa*. Epiphytic especially on *Erica*. So far, only known from Mt. Kilimanjaro, Tanzania.

Selected specimens examined: **Tanzania**, Mt. Kilimanjaro, near Machame Route, fire disturbed subalpine *Erica* vegetation, −3.0854° N 37.2794° E, 3520 masl, 15 June 2017, on *Erica* branches, U. Kaasalainen UK171340t (H). Near Mweka Route, fire disturbed upper montane *Podocarpus* forest now dominated by *Erica excelsa*, −3.1640° N 37.3675° E, 2990 masl, 25 June 2017, on tree trunk, U. Kaasalainen UK171584k (H). Near Umbwe Route, subalpine *Erica trimera* forest, −3.1114° N 37.3183° E, 3500 masl, 18 June 2017, on fallen branch, U. Kaasalainen UK171407j (H). On *Erica*, U. Kaasalainen UK171458k (H).

Notes: Well-developed specimens are easy to distinguish from other *Sticta* species based on their robust, often dark brown appearance, the isidiate scrobiculation at least along lobe margins, and thick dark brown tomentum of the lower surface. Poorly developed specimens may resemble other isidiate species with yellowish–brown upper surface, but can be distinguished on the basis of submarginal, cylindrical isidia.

#### 3.2.6. *Sticta ciliata* Tayl. (Figure 10a)

*Sticta ciliata* has a cyanobacterial main photobiont (*Nostoc*) and rounded lobes with laminal isidia. A detailed description of *S. ciliata* is provided in [20]. In our region, the two sequenced specimens of *S. ciliata* have small, approximately 1–2 cm wide and 0.5–1.5 cm long, loosely adnate, rounded, obovate lobes with abundant, laminal isidia on the grey upper surface. The lobes are revolute making them convex. The lower surface is pale with a tomentose base, but with the tomentum becoming scarce or absent towards the margins, and with flat, variably sized cyphellae. Lobe margins are often ciliate. Chemistry: K−, C−, Pd−.

In our region, four other species with laminal isidia are present: *Sticta aspratilis*, *S. fuliginoides*, *S. fuliginosa*, and *Sticta* sp. B. Our *S. ciliata* specimens are rather few and poorly developed, but Magain and Sérusiaux [20] describe their diagnostic characters: Fresh specimens of *S. ciliata* have delicate and usually ciliate thallus margins, especially in young thalli, and abundant tiny papillae over the cells of the cyphellar membrane, however, the regeneration lobules of all other isidiate species can also have marginal cilia, and the fine anatomy of cyphellae can only be studied from fresh and well-preserved material. In our material, *S. fuliginosa* and *S. aspratilis* usually have larger thalli with a tomentose lower surface and cupuliform to slightly urceolate cyphellae. *Sticta fuliginoides* has a funnel-shaped base and usually occurs on higher elevations than *S. ciliata*. *Sticta* sp. B closely resembles *S. ciliata* in morphology and occurs in similar habitats. However, the material presently available is too scarce to allow a detailed morphological analysis to compare the two species.

Ecology and distribution: In other parts of the world, *S. ciliata* is known from Europe, Macaronesia, and possibly Colombia, where it grows as an epiphyte on tree trunks and on bryophytes, especially in well-preserved, humid forests [20]. The two confirmed specimens in our material were collected from moist montane forest, one from the Sagalla Mountain in the Taita Hills and one from the lower montane forest of Mt. Kilimanjaro. Both specimens were growing epiphytically on tree trunks. *Sticta* sp. B seems to be widely distributed in East Africa and has been collected from Kenya, Tanzania, and Rwanda, from similar moist lower to middle montane forests as *S. ciliata*.

**Figure 10:**
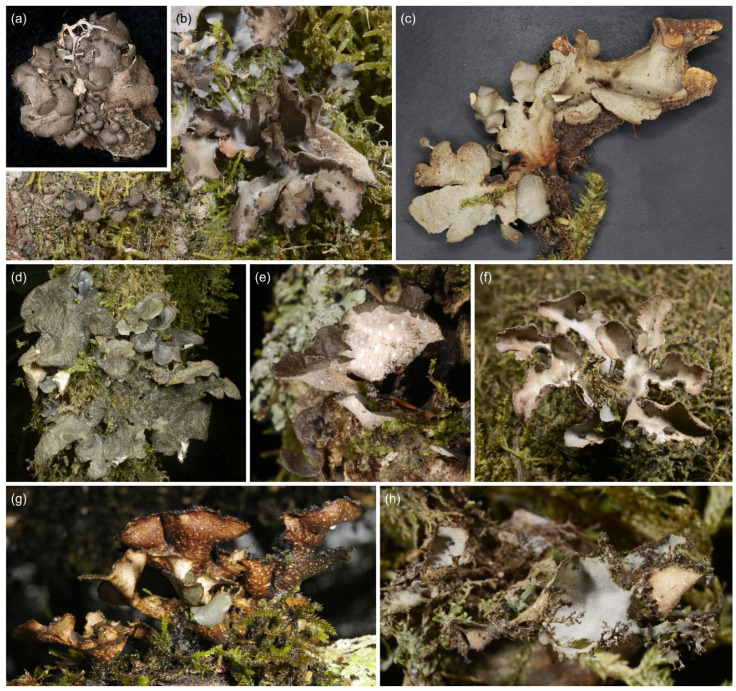
*Sticta ciliata*, *Sticta duplolimbata*, *Sticta fuliginoides*, *Sticta fuliginosa*, and *Sticta marginalis*. (**a**) *Sticta ciliata* from Chawia Forest with small revolute lobes and ciliate margins (UK110570a). (**b**) *Sticta duplolimbata* photographed in situ on Vuria Mountain, showing the elongate lobes with rounded apices and marginal cilia and isidia, and abundantly tomentose lower surface. (**c**) *Sticta fuliginoides* from an upper montane forest on Mt. Kilimanjaro with palmate lobes and a funnel-like base (UK171468d). (**d**,**e**) *Sticta fuliginosa* photographed in situ in the Taita Hills. (**d**) Specimen JR16358 with rounded lobes and abundant laminal isidia. (**e**) Specimen JR09A46 showing the white and abundantly tomentose lower surface. (**f**–**h**) *Sticta marginalis* photographed in situ in the Taita Hills. (**f**) Specimen JR29502 with stipitate, ascending lobes and marginal isidia. (**g**) Note the characteristic etomentose lower surface with inconspicuous cyphellae. (**h**) Delicate marginal isidia and stalked lobules occur commonly especially in ageing thalli.

#### 3.2.7. *Sticta cyanocaperata* Kaasalainen sp. nov. (Figure 11)

Mycobank # MB847046

Species of *Sticta* lichenized with a cyanobacterium and characterized by its light grey to fawn, wrinkled upper surface, flattened marginal isidia, and brown, ridged (visible in thallus cross-section) lower surface without moniliform secondary tomentum.

Type: **Tanzania**, Mt. Kilimanjaro, near the Umbwe Route, middle montane *Ocotea* forest −3.0819° N 37.1819° E, 2650 masl, 20 June 2017, on a fallen branch, U. Kaasalainen UK171480d (H 9237171—holotype).

ITS barcoding marker accession (GenBank): OP999563 (holotype).

Description: *Thallus* rosetteform, (140)200–470 µm thick with thickenings on the lower side clearly visible in the cross-section, closely adnate centrally, loosely adnate marginally. *Lobes* elongate, polytomously branching, up to 5.5 cm long and 2 cm wide; margins often crisped, with darker, mostly flattened isidia, occasionally also with phyllidia. *Upper surface* is usually fawn to yellowish brown, occasionally light grey wavy to slightly wrinkled at least centrally. *Upper cortex* paraplectenchymatous, 30–70 μm and 4–6 cell layers thick, cells 6–13 µm in diam., the first layer(s) more compact. *Photobiont Nostoc*. *Photobiont layer* 30–90 μm thick, with *Nostoc* cells 5–7 μm in diam. *Medulla* 75–300 μm thick, with hyphae 3–4 µm wide. *Cilia* not present. *Isidia* marginal, usually dark brown, glossy, coralloid, flattened. *Lower surface* dark brown at least centrally, may become lighter towards margins, with ridges (may not always be visible under a preparation microscope, but present at least as thickenings in a thallus cross-section), with brown tomentum, lighter margins may be etomentose. *Lower cortex* paraplectenchymatous, (10)30–45 μm and (2)3 cell layers thick, cells 7–12 μm in diam*. Primary tomentum* usually dark brown, composed of agglutinated hyphae, may become entangled and matted centrally, and/or resemble arachnoid secondary tomentum. Moniliform *secondary tomentum* not present. *Rhizines* scattered in groups, dark brown to black, glossy, often with long, tapering, white tip, often hirsute from the lower parts. *Cyphellae* urceolate with a wide opening (up to 1 mm in diam.), pore margin raised or flat, occasionally thickened; cyphellar membrane light yellow, ~20 μm thick, cells rounded, epapillose, 6–7 μm in diam. *Apothecia* or *pycnidia* not seen. *Chemistry*: K−, C−, Pd−.

**Figure 11:**
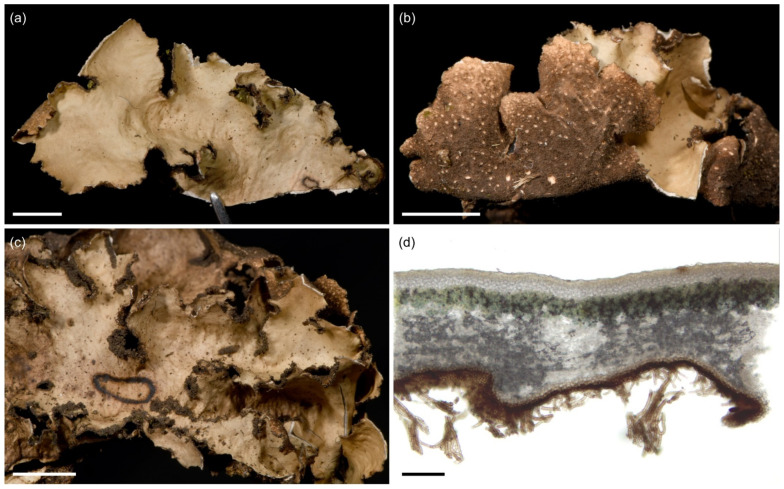
*Sticta cyanocaperata*. (**a**) A fragment of the type specimen UK171480d. (**b**,**c**) Additional thalli part of the same collection as the type: (**b**) Showing the brown tomentum and pale thallus margins. (**c**) Characteristic light yellowish–brown, wrinkled upper surface and dark, flattened, marginal isidia. (**d**) Thallus cross section showing the uneven lower surface (UK170912a) and the lack of moniliform secondary tomentum. Scales 0.5 cm in (**a**–**c**), 100 µm in (**d**).

Etymology: The specific epithet refers to the cyanobacterial primary photobiont and characteristic wrinkles on the upper and lower surfaces, and to the close phylogenetic affiliation to *Sticta caperata*.

Ecology and distribution: *Sticta cyanocaperata* is common in the middle montane forests on Mt. Kilimanjaro (2270–2650 masl). Epiphytic on canopy branches, tree trunks, and lianas. So far, *S. cyanocaperata* is only known from Mt. Kilimanjaro, Tanzania.

Selected specimens examined: **Tanzania**, Mt. Kilimanjaro, near Marangu Route, disturbed middle montane *Ocotea* forest, −3.1207° N 37.3057° E, 2370 masl, 14 March 2017, on fallen branch, U. Kaasalainen UK170912a (H). U. Kaasalainen UK170912e (H). Near Maua Route, disturbed middle montane *Ocotea* forest, −3.1319° N 37.2717° E, 2270 masl, 29 May 2017, on liana, U. Kaasalainen UK171182c (H). Near Umbwe Route, middle montane *Ocotea* forest, −3.0824° N 37.1811° E, 2540 masl, 20 June 2017, on tree trunk, U. Kaasalainen UK171495f (H).

Notes: In shady habitats, *S. cyanocaperata* may have a rather different appearance in having a light grey upper surface, thin thallus, few isidia, and poorly developed lower side tomentum. However, such specimens can still be identified on the basis of the characteristic lower side ridges and lack of moniliform secondary tomentum. The closely related *S. caperata* most commonly has a green algal primary photobiont. The cyanomorph of *S. caperata* reported from Réunion [25] differs from *S. cyanocaperata* in having a marbled upper surface. *Sticta xanthotropa*, previously reported from East Africa [12], is distinguished for example based on the pale cream-colored to yellowish lower surface [32]. The most closely resembling species in the region with similar flattened marginal isidia and yellow–brown upper surface is *S. andina* which can be recognized due to the instant and strong K+ yellow reaction of the medulla.

#### 3.2.8. *Sticta duplolimbata* (Hue) Vain. (Figure 10b)

*Sticta duplolimbata* has a cyanobacterial main photobiont (*Nostoc*) and marginal isidia. A detailed description of the species is provided in [59]. In our region, the species is characterized by loosely adnate, elongate, and most often light grey lobes with rounded apices, dark, mainly marginal cylindrical to coralloid isidia, and dark marginal cilia which, however, are not always present. The lower side is abundantly tomentose, with tomentum reaching the lobe margins, and usually pale but sometimes even dark brown. Cyphellae are conspicuous, raised, and distinctly urceolate with a small opening. Chemistry: K−, C−, Pd−.

*Sticta duplolimbata* is easy to distinguish from the other *Sticta* species in our region, especially by the dark cilia usually present at the rounded lobe apices and distinct, urceolate cyphellae. Galloway [59] mentions that *S. duplolimbata* has “lobes arising from short stalk”; however, this is not visible in most of our specimens, which are usually attached to their substrate by the tomentum of the central parts of the lower surface.

Ecology and distribution: In other parts of the world, *S. duplolimbata* is mainly known from the Western Pacific region [59]. Abundant on tree trunks, climbers, and canopy branches in lower and middle montane forests on Mt. Kilimanjaro, with fewer specimens from upper montane forests, Chagga home gardens, and moist montane forests of the Taita Hills (1800–3060 masl).

#### 3.2.9. *Sticta fuliginoides* Magain and Sérus. (Figure 10c)

*Sticta fuliginoides* has a cyanobacterial main photobiont (*Nostoc*) and obovate lobes with laminal isidia. A detailed description of the species is provided in [20]. In our region, *S. fuliginoides* is characterized by the obovate lobes, ascending from a single attachment point. The lobes are usually up to 2.5 cm in diam and have a funnel-like base. The upper surface is grey, brown or yellowish–brown, smooth to strongly reticulate especially in ageing thalli, and with dark, mainly laminal, cylindrical to coralloid isidia, often developing into stalked lobules. The lower surface is white or beige, with abundant tomentum near the attachment point, but often etomentose towards the margins. Cyphellae are variable in size, usually urceolate and dome-like and raised especially towards the thallus margin, and if cupuliform, with a distinctly raised margin. Chemistry: K−, C−, Pd−.

Poorly developed thalli and/or specimens collected from suboptimal habitats may often have only one obovate-palmate ascending lobe with laminal isidia, and a mostly etomentose lower surface with pronounced cyphellae. In well-developed thalli, the ascending lobes usually have a funnel-shaped base quite distinct from those of other *Sticta* species with laminal isidia. Based on our phylogenetic analysis, three specimens, closely resembling *S. fuliginoides* in overall morphology, represent an additional undescribed taxon *Sticta* sp. A (Figure 3). However, the material presently at hand is insufficient for properly assessing the morphological characteristics of that species.

Ecology and distribution: In other parts of the world (Europe, Macaronesia, North America, Colombia) *S. fuliginoides* grows on mossy trees and rocks in humid forests and parks [20]. In our region it is an abundant epiphyte on tree trunks, canopy branches, and climbers, especially in upper montane forests on Mt. Kilimanjaro, but occurring in middle montane to subalpine habitats as well (2470–3520 masl).

#### 3.2.10. *Sticta fuliginosa* (Hoffm.) Ach. (Figure 10d,e)

*Sticta fuliginosa* has a cyanobacterial main photobiont (*Nostoc*) and laminal isidia. A detailed description of the species and a discussion of differences between *S. fuliginosa*, *S. ciliata*, and *S. fuliginoides* are provided in [20]. In our region, *S. fuliginosa* is characterized by rounded palmate-obovate lobes, usually up to 5 cm in diam., with an uneven, most often greyish–brown upper surface and darker laminal isidia. The lower surface is pale with cupuliform to slightly urceolate cyphellae, and with a pale primary tomentum, but usually without a moniliform secondary tomentum. Chemistry: K−, C−, Pd−.

Of the other species with laminal isidia in our region, *S. fuliginosa* mostly resembles *S. aspratilis* which, however, has a well-developed arachnoid secondary tomentum on the lower surface, often making also the primary tomentum appear “furry”. Small thalli of *S. fuliginosa* often have dark brown, rounded lobes with ciliate and occasionally revolute margins very similar to those of *S. ciliata* agg. *Sticta ciliata* is usually much smaller and according to Magain and Sérusiaux [20], fresh specimens of *S. ciliata* and *S. fuliginoides* both have abundant papillae on the cells of cyphellar membrane, which do not occur in *S. fuliginosa*.

Ecology and distribution: *Sticta fuliginosa* is believed to be widely distributed in both hemispheres [20,60]. In our region, it is common in the Taita Hills forests and occurs on Mt. Kasigau. On Mt. Kilimanjaro it is common especially on canopy branches in middle montane forests but is also found in the lower montane to upper montane zones, including the Chagga home gardens (1840–2880 masl).

#### 3.2.11. *Sticta marginalis* Bory (Figure 10f–h)

*Sticta marginalis* has a cyanobacterial main photobiont (*Nostoc*) and marginal isidia that often develop into stalked lobules. Thallus lobes are clearly stipitate, palmate, and ascending from a single attachment point. The upper surface is smooth, usually light grey with a yellow tinge. Isidia are dark, mainly marginal, cylindrical to coralloid, and often develop into characteristic stalked lobules especially in ageing thalli. Lower surface is light to dark brown, often with some yellow coloring, usually without tomentum or with a limited amount of short velvety hair; cyphellae are small and flat. Apothecia are not present in our material, but in the description of *S. marginalis* from Réunion, the apothecia are described submarginal and the ascospores brown, 1–3-septate, 40–48 × 8 µm [61]. Chemistry: K−, C−, Pd−.

Sticta marginalis is very characteristic looking and easily distinguished from the other *Sticta* species in the region based on the stipitate, palmate, ascending lobes with isidiate-lobulate margins, mostly naked lower surface, and flat cyphellae.

Ecology and distribution: In other parts of the world, *Sticta marginalis* is known from its type location Réunion and from Madagascar [61,62,63]. In our region, *S. marginalis* often grows as an epiphyte on tree trunks, but occasionally also on decaying wood and rock. It is common in moist lower and middle montane forests of Mt. Kilimanjaro, the Taita Hills, and Mt. Kasigau (1450–2470 masl).

Note: *Sticta marginalis* was described from Réunion [61] and has not previously been reported from the African continent. Another stipitate species *S. orbicularis*, originally described from Java in Indonesia [64], has previously been reported to occur in East Africa [12,28,30]. *Sticta marginalis* has even been suggested to be synonymous to *S. orbicularis* [65], and also Swinscow and Krog [12] noted that the taxon needs critical study and that *S. pusilla* Meissner may be its correct name. No DNA data is available from *S. orbicularis* and a description of the type material mentions that the material consists of only two immature specimens [64].

#### 3.2.12. *Sticta munda* Kaasalainen sp. nov. (Figure 12)

Mycobank# MB847047

Species of *Sticta* lichenized with a cyanobacterium and characterized by palmate lobes with light grey upper surface, lack of symbiotic propagules, and submarginal apothecia with nodular margins and 3-septate, 30–40 µm long ascospores.

Type: **Tanzania**, Mt. Kilimanjaro, near the Mweka Route, upper montane secondary forest with *Erica excelsa*, −3.1640° N 37.3675° E, 2990 masl, 25 June 2017, on a tree trunk, U. Kaasalainen UK171584u (H 9237172—holotype).

ITS barcoding marker accession (GenBank): OP999600 (holotype).

Description: *Thallus* loosely adnate, often rosetteform, (120)150–180(210) µm thick and up to 5(7) cm in diam., attached from the center of the lower side or, more often, consisting of a single ascending, palmate lobe attached to substrate from its base. *Lobes* relatively thin, palmate, 1.5–3(4) cm in diam., usually isodiametric or wider than long, with rounded margins. *Upper surface* light grey or occasionally brownish towards margins, smooth to slightly wavy or foveolate. *Upper cortex* paraplectenchymatous, (20)25–35(50) μm and 3–4(6) cell layers thick, composed of isodiametric cells 7–11 μm in diam., the cells in the first layer(s) occasionally slightly flattened and/or smaller. *Photobiont Nostoc*. *Photobiont layer* (30)35–50(70) μm thick, with *Nostoc* cells 5–8 μm in diam. *Medulla* (35)40–75 μm thick, with hyphae 2–4 µm wide. *Cilia*, i*sidia, soredia*, and *phyllidia* absent. *Lower surface* cream colored to light brown, smooth, with abundant tomentum. *Lower cortex* paraplectenchymatous, 15–35(40) μm and 2–3(4) cell layers thick, with isodiametric to slightly oblong cells 6–13 μm in diam*. Primary tomentum* with brown and agglutinated stems, becoming lighter and squarrose towards the tips. *Secondary tomentum* white, arachnoid, composed of moniliform assemblages. *Rhizines* not present. *Cyphellae* (40)50–200/cm^2^, cupuliform and rounded with a clearly raised margin when small to medium sized, irregular and slightly urceolate when large; pore rarely >0.8 mm in diam., often surrounded by a darker ring; cyphellar membrane white, cells rounded, epapillose, 6–9 μm in diam. *Apothecia* common, submarginal to laminal, up to 2 mm in diam.; disc light reddish to dark brown; margin light brown with darker brown nodules. *Exciple margin* 120–140 μm wide. *Epithecium* 10–15(30) μm thick, orange–brown. *Hymenium* (80)90–110 μm thick. *Hypothecium* (45)80–95 μm thick, orange–brown. *Ascospores* fusiform, colorless, 3-septate, 30–38(43) × (5)6–9 µm. *Pycnidia* not seen. *Chemistry*: K−, C−, Pd−.

**Figure 12:**
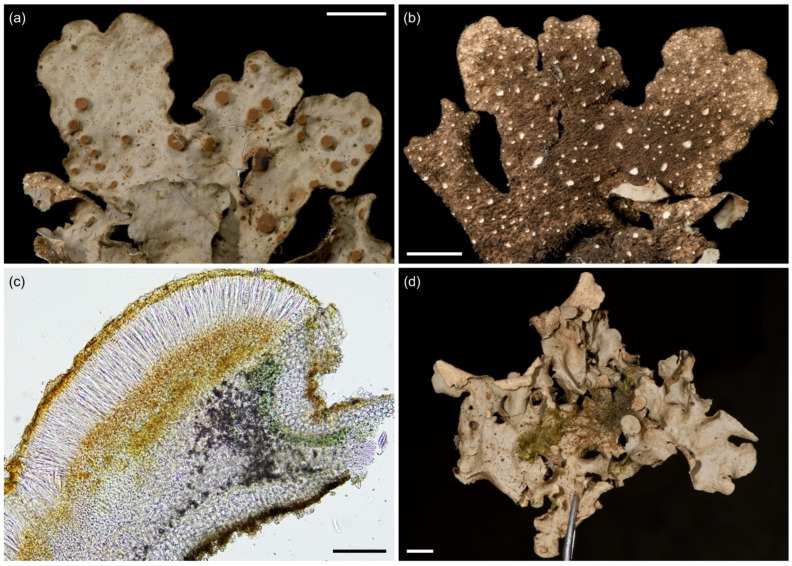
*Sticta munda*. (**a**–**c**) Type specimen UK171584u. (**a**,**b**) Typical single lobed, palmate, and abundantly fertile specimen seen from the upper and lower side. (**c**) A cross section of apothecium. (**d**) Specimen UK171582i with a rosetteform thallus. Scales 0.5 cm in (**a**), (**b**), and (**d**), 100 µm in (**c**).

Etymology: The specific epithet refers to the simple and elegant form of the species.

Ecology and distribution: *Sticta munda* is quite rare in the middle and upper montane forests on Mt. Kilimanjaro (2220–2990 masl). Epiphytic on canopy branches and tree trunks. So far, only known from Mt. Kilimanjaro, Tanzania.

Selected specimens examined: **Tanzania**, Mt. Kilimanjaro, near the Marangu route, upper montane *Podocarpus* forest, −3.1044° N 37.3046° E, 2800 masl, 13 March 2017, on a fallen branch, U. Kaasalainen UK170888c (H). Near the Mweka route, −3.1101° N 37.2130° E, 2470 masl, 24 June 2017, on a fallen branch, U. Kaasalainen UK171508j (H), U. Kaasalainen UK171510k (H). Upper montane secondary forest with *Erica excelsa*, −3.1640° N 37.3675° E, 2990 masl, 25 June 2017, on a tree trunk, U. Kaasalainen UK171582i (H). Near the Umbwe route, disturbed middle montane *Ocotea* forest, −3.0903° N 37.1724° E, 2220 masl, 20 June 2017, on a fallen branch, U. Kaasalainen UK171497d (H).

Notes: The six specimens of *S. munda* sequenced represented three different ITS variants. Although all the specimens formed a well-supported monophyletic clade, there are some morphological differences between specimens representing the different ITS variants. *Sticta munda* resembles *S. ambavillaria* from Réunion, which, however, is distinguished by the phylogenetic analysis (Figure 2). *Sticta ambavillaria* also has longer ascospores ((42)44.5–50 μm [33]) than *S. munda*, which was apparently also noticed by Swinscow and Krog [12] who reported short ascospores (30–40 × 6–10 µm) for their *S. ambavillaria* specimens collected from East Africa. Additionally, fertile specimens of *S. umbilicariiformis* and *S. aspratilis* can resemble *S. munda*, but they have one-septate ascospores and thicker lobes, which are often brown instead of grey and more distinctly foveolate.

#### 3.2.13. *Sticta sublimbata* (J. Steiner) Swinscow and Krog (Figure 13a,b)

*Sticta sublimbata* has a cyanobacterial main photobiont (*Nostoc*) and marginal soralia. A detailed description of East African material is provided in [12]. The rosetteform thallus has adnate or loosely adnate, elongate more or less linear and narrow (usually < 1 cm wide) lobes with rounded apices that are often also revolute when dry. The upper surface is usually light leather brown to grey but may also be dark brown. The lower surface is light to dark brown, and usually has scarce, short tomentum, but also densely tomentose forms are quite common. Cyphellae are white with open, raised margins. Marginal linear soralia are almost always present and produce farinose to granular soredia, and sometimes also form aggregates resembling isidia. Apothecia not seen. Chemistry: K−, C−, Pd−.

In our region, *S. sublimbata* is the only sorediate species in lower montane forests and woodland below 2500 masl. *Sticta umbilicariiformis*, which is common in the upper montane and subalpine zones, can occasionally appear sorediate, but usually has much thicker and wider (several centimeters wide) lobes and an abundantly tomentose lower surface.

Ecology and distribution: In addition to Africa, *S. sublimbata* is known from Australia, New Zealand, and southern South America [59]. In our region, *S. sublimbata* is an abundant and common epiphyte especially on tree trunks, but it also grows among bryophytes on cliffs and other rock surfaces. *Sticta sublimbata* is especially common in the lower montane forests of the Taita Hills, but also occurs on Mt. Kasigau and Mt. Kilimanjaro, mainly below 2000 masl. It may even benefit from human activity as it seems most abundant in moderately disturbed habitats.

**Figure 13:**
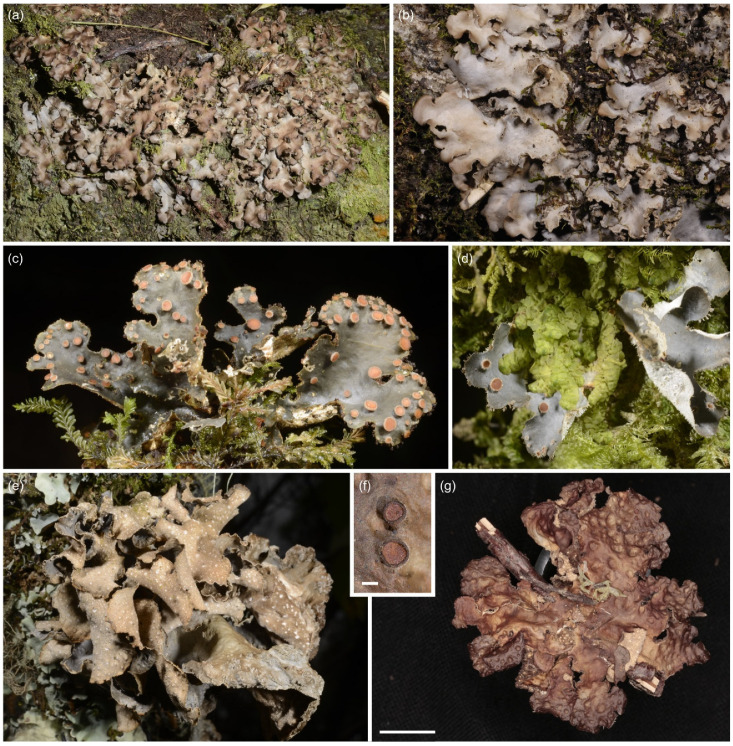
*Sticta sublimbata*, *Sticta tomentosa*, and *Sticta umbilicariiformis*. (**a**,**b**) *Sticta sublimbata*. (**a**) Large and adnate thallus of specimen JR16275 photographed in situ on Sagalla Mountain. (**b**) Specimen JRCA2 photographed in situ in Ngangao Forest displaying characteristic rounded lobes and marginal soralia. (**c**,**d**) *Sticta tomentosa* photographed in situ on Vuria Mountain. (**c**) Specimen JR16368 with palmate lobes and abundant apothecia. (**d**) Specimen JRA14357 showing the lobe margins with tufts of hair, apothecia with white hairs on the margin, and white lower surface with prominent cyphellae. (**e**–**g**) *Sticta umbilicariiformis*. (**e**) Specimen JR16102 photographed in situ in the ericaceous zone on Mt. Elgon with characteristic pustules on thallus margins and submarginal lamina. (**f**,**g**) Fertile specimen UK171411c with strongly foveolate upper surface and tomentose apothecial margins. Scales 0.5 mm in (**f**), 1 cm in (**g**).

#### 3.2.14. *Sticta tomentosa* (Sw.) Ach. (Figure 13c,d)

*Sticta tomentosa* has a cyanobacterial main photobiont (*Nostoc*) and palmate lobes without symbiotic propagules, but usually with apothecia. A detailed description of *S. tomentosa* is provided in [66] and, based on East African material, in [12]. Our material of *S. tomentosa* have light grey, palmate lobes ascending clearly from one attachment point, and with tufts of hairs projecting from the lobe margins. The lower surface is white with abundant white tomentum and has prominent, dome-like cyphellae. *Sticta tomentosa* does not produce isidia, soredia nor phyllidia, but is almost always fertile. The apothecia are mainly submarginal with long white hairs on the margins. Ascospores are fusiform, colorless, 3-septate, 39–50 × 7–11 µm (30–50 × 6–10 µm in [12]). Chemistry: K−, C−, Pd−.

*Sticta tomentosa* is the only species in the studied lower montane forests that is commonly fertile and does not produce any type of symbiotic propagules. Easily distinguished from other fertile species in the region based on the thick white hairs on apothecial margin. The apothecial margins of other species are pubescent at most, and this feature is usually only seen in specimens collected from high-elevation habitats. Furthermore, the three-septate ascospores of *S. tomentosa* are larger than those of any other species in the region. The ascospores in the type specimen of *S. tomentosa* were measured to be 27.5–33.5(−36) × 5.5–8.5 µm [66], which is considerably less than in our material. Previously, also Swinscow and Krog [12] reported a relatively large ascospore size (30–40(50) × 6–10 um) from East Africa.

Ecology and distribution: *Sticta tomentosa* is a pantropical species found especially in undisturbed, middle to high elevation rainforest habitats [60]. In our region, *S. tomentosa* is not very common and grows as an epiphyte on tree trunks. It can occasionally be locally abundant in lower montane forests, and also occurs in middle and upper montane forests (1650–3060 masl) in the Taita Hills and on Mt. Kilimanjaro.

#### 3.2.15. *Sticta umbilicariiformis* Hochst. ex Flot. (Figure 13e–g)

*Sticta umbilicariiformis* has a cyanobacterial main photobiont (*Nostoc*) and typically many marginal pustules which can sometimes make it appear sorediate. Thalli of *S. umbilicariiformis* are often quite large with thick (usually 200–350 µm), wavy to foveolate, loosely adnate to ascending lobes 3–5 cm long and 2–4 cm wide. The upper surface is greyish brown with congested marginal, and often also laminal pustules that may occasionally appear sorediate. The lower surface is cream colored or, more rarely, brown, and thickly tomentose. Primary tomentum has brown and agglutinated stems and squarrose, white tips. Secondary tomentum is white and arachnoid, composed of moniliform assemblages. Cyphellae are slightly urceolate with a relatively wide opening (up to 1 mm in diam.) and raised margins, the pore is often surrounded by a darker ring.

Also fertile specimens of *S. umbilicariiformis* are common and usually lack symbiotic propagules. The thallus lobes of palmate fertile specimens are often thinner than the lobes of pustulate specimens and vary from wavy to strongly foveolate. Apothecia are submarginal and laminal, up to 2(3) mm wide, with brown disks and, occasionally, pubescent margins patterned with brown nodules. Ascospores are colorless, fusiform, 1-septate, 31–40 × 6.5–8 µm. Chemistry: K−, C−, Pd−.

Pustular thalli of *S. umbilicariiformis* are easily distinguished from other species in the region merely based on their habit. The only vaguely similar species is *S. sublimbata* which, however, produces true marginal soralia, has adnate and narrow lobes, and mainly occurs below 2000 masl. The characters that help to distinguish fertile specimens of *S. umbilicariiformis* from fertile specimens of *S. aspratilis* and *S. munda* are described under those species.

Ecology and distribution: *Sticta umbilicariiformis* is presently confirmed only from East Africa, but might have a much wider distribution reaching North America and Australia [20]. In our region, *S. umbilicariiformis* is common and abundant in the upper montane and subalpine zones on Mt. Kilimanjaro, and also occurs in middle montane zone (between 2540–3800 masl). It is by far the most abundant *Sticta* species in the subalpine zone, and was also collected from the *Erica* zone on Mt. Elgon. In the upper montane forest, *S. umbilicariiformis* mainly grows epiphytically on tree trunks and branches, in the subalpine zone also on rock and soil among bryophytes.

Notes: The phylogeny within the *S. umbilicariiformis* clade remains poorly resolved even in the more detailed analysis (Figure 2). Almost all pustular specimens represent the same ITS variant (umbilicariiformis1), closely related to a sequence from Rwanda (KT281697). The other ITS variants (umbilicariiformis2–5) are mainly from specimens without pustules, but which often have apothecia. The type specimen of *S. umbilicariiformis* (H-Nyl 33835; originally described in [67], lectotype designated in [20]) has both pustules and apothecia on the same thallus and the only fertile specimen of *S. umbilicariiformis* in our material that also has pustules belongs to ITS variant group umbilicariiformis4.

### 3.3. Sticta Diversity in the Montane Ecosystems of East Africa

**Figure 14:**
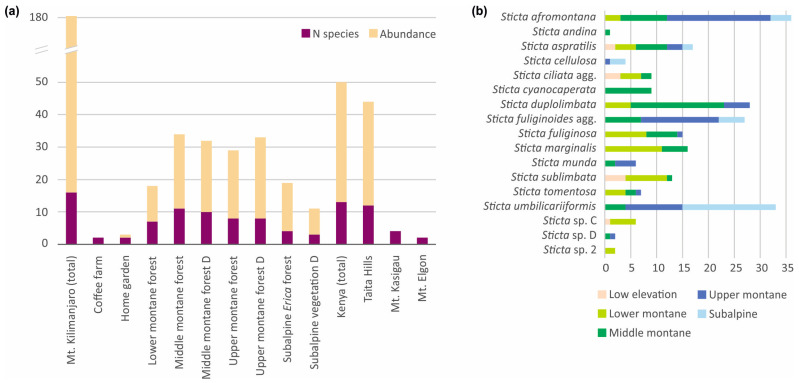
Diversity and abundance of the genus *Sticta* in the study region. (**a**) Number of species (dark red) and abundance (light orange) in the different habitats and locations. D = disturbed. (**b**) The abundance of the *Sticta* species in different elevation zones and habitat types. Low elevation habitats: dry woodland on Mt. Kasigau, the Maktau Hill, Shomoto Hill in the Taita Hills, and coffee farm and home garden habitats on Mt. Kilimanjaro; Lower montane forests: most evergreen forests in the Taita Hills and on Mt. Kasigau, and lower montane forest on Mt. Kilimanjaro. Middle montane forests: middle montane forests on Mt. Kilimanjaro and on Vuria Mountain in the Taita Hills. Upper montane forest: such forests on Mt. Kilimanjaro. Subalpine: subalpine habitats on Mt. Kilimanjaro and Mt. Elgon. *Sticta ciliata* agg. includes specimens of *S. ciliata* and *Sticta* sp. B; *Sticta fuliginoides* agg. includes specimens of *S. fuliginoides* and *Sticta* sp. A.

Altogether 16 species of *Sticta* were collected from Mt. Kilimanjaro, of which *S. cyanocaperata*, *S. fuliginoides*, *S. munda*, *Sticta* sp. A, and *Sticta* sp. D were not found from other locations. The Taita Hills had 12 species, of which *S. andina* and *Sticta* sp. C (*weigelii* agg.) were not collected from the other locations. Mt. Kasigau had four species, and two species were collected from Mt. Elgon (Figure 14). A clear majority of all *Sticta* specimens were collected from montane forests, with not a single observation from lowland savannas or alpine *Helichrysum* heaths, or from agricultural or grassland habitats. The species diversity was highest in middle montane forests (Figure 14a). *Sticta sublimbata* was by far the most common *Sticta* species in the relatively open low elevation habitats. *Sticta afromontana*, *S. duplolimbata*, *S. marginalis*, and *S. fuliginosa* were common in lower to middle elevation forests, while upper montane and subalpine habitats were dominated by *S. umbilicariiformis*, *S. afromontana*, and *S. fuliginoides* (Figure 14b).

## 4. Discussion

A total of 19 *Sticta* species, including five species new to science, were found from the studied mountains in Kenya and Tanzania, and at least five other new species remain to be described pending more material. Of these species, only *S. fuliginosa*, *S. sublimbata*, *S. tomentosa*, and *S. umbilicariiformis* were known to be present in Kenya and/or Tanzania based on previous reports [12,20,28,29,30,31]. *Sticta ciliata* and *S. duplolimbata* had been previously reported from Rwanda [20] and are now confirmed to also occur in Kenya and Tanzania. Additionally, *S. marginalis* and *S. fuliginoides* are here reported as new for Kenya and Tanzania, and *S. andina* as new for Kenya.

Five species were described as new to science: *Sticta afromontana*, *S. aspratilis*, *S. cellulosa*, *S. cyanocaperata*, and *S. munda*, the three last mentioned of which have so far only been collected from Mt. Kilimanjaro. Additionally, five putative species, *Sticta* sp. A in the *Sticta fuliginoides* aggregate, *Sticta* sp. B in the *Sticta ciliata* aggregate, *Sticta* sp. C in the *Sticta weigelii* aggregate, *Sticta* sp. D, and *Sticta* sp. 2 were well resolved in the phylogenetic tree, but are not yet described due to insufficient material. In contrast to previous reports, it seems unlikely that *S. ambavillaria*, *S. cyphellulata*, *S. limbata*, *S. kunthii*, *S. orbicularis*, *S. papyracea/variabilis*, *S. weigelii*, or *S. xanthotropa* would occur in East Africa, as specimens with similar thallus morphologies are here shown to represent other species. Our study confirms the presence of 14 *Sticta* species in Kenya and 17 in Tanzania. Additionally, *S. dichotoma* and *Sticta* sp. 2 are expected to occur in Tanzania based on previous observations [12,28,30], raising the current total number of *Sticta* species in Tanzania to 19. A short synopsis of all *Sticta* species reported from East Africa is provided in Table 1.

Several previous studies have demonstrated that many of the “traditional” *Sticta* species, such as *S. fuliginosa* and *S. weigelii*, include taxa that belong to several different evolutionary lineages [19,20,21,68]. The previously reported *S. fuliginosa* is accompanied by several other taxa with laminal isidia also in East Africa: *Sticta ciliata, S. fuliginoides*, *S. aspratilis*, *Sticta* sp. B, and *Sticta* sp. A. Of these, *S. fuliginoides* and *Sticta* sp. A belong to the same large clade (Figure 3), are morphologically quite similar, and occur in similar habitats. The same applies to *S. ciliata* and *Sticta* sp. B. This suggests that there may still be significant undetected diversity hiding under the name *S. fuliginosa*, both in East Africa and globally. 

Several *Sticta* species belonging to the *S. weigelii* morphodeme, i.e., those with elongate lobes and marginal isidia [21], were found from our region: *Sticta andina*, *S. afromontana*, *S. cyanocaperata*, and *Sticta* sp. C. Most of these have flattened isidia, but the isidia of *Sticta* sp. C are cylindrical, resembling those of *S. weigelii* s. str. [58,69]. However, *Sticta* sp. C does not have a color reaction with K, in contrast to the K+ yellow of *S. weigelii* s. str. [69], giving further support to our phylogenetic analysis, which indicated that *S. weigelii* s. str. has not been collected from East Africa. This supports the notion that *S. weigelii* s. str. may be restricted to the Neotropics [21,58]. In East Africa, *Sticta* specimens with flattened marginal isidia have previously been assigned to *S. xanthotropa* [12], a species that also mainly occurs in the Americas [32]. However, our results indicate that none of the East African species with flattened isidia actually represent *S. xanthotropa*: *Sticta andina* has a strong K+ yellow color reaction, while *S. xanthotropa* is K− [32]; *Sticta afromontana* has a robust thallus, while *S. xanthotropa* is described as “papery thin”, and the spores of *S. afromontana* are larger than those of *S. xanthotropa* [32]; *Sticta cyanocaperata* has a very dark lower surface, while it is pale in *S. xanthotropa* [32]. Furthermore, all the aforementioned species are mainly epiphytic, while *S. xanthotropa* has been reported to mainly grow on rocks and soil [32,70].

The group of species that have previously been reported from East Africa, but the presence of which we were not able to confirm include *S. cyphellulata*, *S. orbicularis, S. limbata, S. ambavillaria* and *S. kunthii* [12,26,27,28,30]. Our specimens morphologically matching the previous reports of *S. orbicularis* and *S. cyphellulata* were assigned to *S. marginalis* and *S. duplolimbata*, while the previously reported *S. limbata* represents *S. umbilicariiformis*, as already suggested by Magain and Sérusiaux [20]. *Sticta ambavillaria* and *S. kunthii* are both fertile species devoid of symbiotic propagules, and the previous reports from East Africa likely refer to *S. munda* and/or fertile specimens of *S. aspratilis* or *S. umbilicariiformis*. Based on our phylogenetic analyses, a specimen of *S. ambavillaria* (JQ735978) from Réunion from where the species was described [63], does not group with any of the East African specimens. Furthermore, already Swinscow and Krog [12] reported that the ascospores of East African specimens were shorter than what has been described from the type of *S. ambavillaria* [33]. *Sticta kunthii* was described from Peru and appears to have a mainly neotropical distribution [32,33]. It has been reported only once from East Africa, from an upper montane forest on Mt. Kenya. The specimen(s) were described to have “a thallus surface with numerous depressions, sometimes appearing almost pitted, a pale lower tomentum with medium-sized cyphellae, and apothecia with short marginal hairs” [26], which corresponds well with some fertile specimens of *S. aspratilis* and *S. umbilicariiformis*. However, the apothecia of these species are submarginal or laminal while those of *S. kunthii* are mainly marginal [32,33]. Furthermore, the apothecial margins of *S. kunthii* are distinctly hairy with long bundles of silky, white hairs [33], while those of East African specimens have only velvety stubble. No sequences have as yet been published from *S. kunthii*, but Moncada et al. [19] placed the species within the *S. kunthii* clade based on morphological evidence; in our material the only taxon belonging to that clade is the isidiate *Sticta* sp. D.

*Sticta dichotoma* and *S. papyracea/variabilis* are the only two species with green algae as the main photobiont previously reported to occur in East Africa, both from Tanzania where they are said to be rare [12,28,30]. While we did not find any specimens matching the description of *S. dichotoma*, the species may well be present in montane forests of Tanzania [12,28,30]. Our four *Sticta* specimens with a green algal photobiont all have marginal phyllidia, and thus correspond morphologically with *S. papyracea* [12]. *Sticta papyracea* and the synonymous *S. variabilis* were both originally described from Réunion [33,71]. However, in the phylogenetic analysis our specimens did not group together with *S. variabilis* from Réunion (Figure 5), but instead formed a clade with specimens of “*Sticta* sp. 2” by Simon et al. [25] collected from Madagascar. This putative species was described to have a green algal photobiont, elongated and dichotomously branching lobes, and apothecia, but lack lobules and phyllidia [25]. Hence, it seems quite possible, that our specimens represent yet another undescribed species. However, more collections are needed before definite conclusions can be made.

In the regions examined, the diversity of *Sticta* species was highest in indigenous moist forests of the middle montane zone. Two other Peltigeralean lichen genera, *Leptogium* and *Peltigera*, exhibited slightly different diversity patterns, with the highest diversity of *Leptogium* species recorded from moist lower montane forests and that of *Peltigera* species from the upper montane zone [13,17]. In comparison to the approximately 20 species of *Sticta* present in the study region, the genus *Leptogium* is much more diverse with possibly over 70 species in the region [13], while only 8 species of *Peltigera* have so far been collected from Mt. Kilimanjaro [17]. When comparing the natural and disturbed habitat types on Mt. Kilimanjaro, the number of *Sticta* species was usually at least slightly lower in disturbed habitats. Similar patterns has previously been reported also for *Leptogium* and *Peltigera* [13,17]. Even while the effects and degree of disturbance varied considerably between different habitat types, all the disturbed habitats tended to be at least slightly more open, and often considerably so, than the unaltered habitat types [34], with probable effects to illumination conditions, temperature, and humidity. A similar trend of decreasing lichen species diversity with increasing habitat disturbance has been observed also in previous studies [72,73,74]. Especially shade-adapted cyanolichens are easily negatively affected by disturbance, and these effects on total species diversity are not necessarily compensated by a concurrent increase in the number of heliophytic species [72]. 

## 5. Conclusions

At least 20 species of *Sticta* have now been confirmed to occur in East Africa, which is almost double to that known before. Only four of the presently accepted species are identified under the same species names that have been used in previous studies from the region, highlighting the general need of taxonomic revisions of lichenized fungi in Africa. The overall diversity of *Sticta* in East Africa is substantially higher than previously known, but seems to be lower than what has recently been recorded from some mountain regions in the Neotropics. Interestingly, even though our collections originated in relatively few mountain regions of Kenya and Tanzania, we still managed to collect specimens corresponding to almost all morphological species previously reported from East Africa. Both the relatively high number of novel taxa detected, and the percentage of species represented by only a few specimens and/or found from single localities, indicates that more comprehensive sampling will undoubtedly reveal further diversity in the genus *Sticta* in East Africa.

## Figures and Tables

**Table 1:** Summary of the genus *Sticta* in East Africa, including Ethiopia (E), Kenya (K), Rwanda (R), Tanzania (T), and Uganda (U). Distributions are reported according to our observations and previous studies listed in References. The ecological notes are based on our observations.

Species	Distribution	Ecology	Comments	References
*Sticta afromontana* sp. nov.	K*, T*	Common especially in middle and upper montane forests.		
*Sticta ambavillaria*	-	Not found in this study.	Previous reports may represent *S. munda*.	[12,27,28,29,30]
*Sticta andina*	K*	Middle montane forest (single observation).		
*Sticta aspratilis* sp. nov.	K*, T*	Lower montane to subalpine zones.		
*Sticta cellulosa* sp. nov.	T*	Rare in upper montane and subalpine zones.		
*Sticta ciliata*	K*, T*	Rare in lower montane forest zone.	Previous report from Rwanda [20] represents *Sticta* sp. B.	
*Sticta cyanocaperata* sp. nov.	T*	Uncommon in middle montane forest.		
*Sticta cyphellulata*	-	Not found in this study.	Previous reports may represent *S. duplolimbata* and/or *S. marginalis*.	[12,28,30]
*Sticta dichotoma*	T	Not found in this study.		[12,28,30]
*Sticta duplolimbata*	K*, T*, R	Common in lower and middle montane forests.		[20]
*Sticta fuliginoides*	T*	Common especially in upper montane forests.		
*Sticta fuliginosa*	K, T, R	Common in lower and middle montane forests.	Some previous reports may represent other species of *S. fuliginosa* morphodeme.	[12,20,28,29,30,31]
*Sticta kunthii*	-	Not found in this study.	Previous report may represent *S. umbilicariiformis* and/or *S. aspratilis*.	[26]
*Sticta limbata*	-	Not found in this study.	Previous reports represent *S. umbilicariiformis* [20].	[12,27]
*Sticta marginalis*	K*, T*, U*	Common in lower and middle montane forests.	Presence in Uganda based on previous reports of *S. orbicularis* [12,28,30].	
*Sticta munda* sp. nov.	T*	Rare in middle and upper montane forests.		
*Sticta orbicularis*	-	Not found in this study.	Previous reports may represent *S. marginalis*.	[12,28,30]
*Sticta papyracea/variabilis*	-	Not found in this study.	*Sticta papyracea* and *S. variabilis* are synonymous [33]. The previous reports may represent *Sticta* sp. 2.	[12,30]
*Sticta sublimbata*	E, K, T, U	Common in low elevation and lower montane habitats.		[12,30,31]
*Sticta tomentosa*	K, T, U	Lower and middle montane forests.		[12,28,30,31]
*Sticta umbilicariiformis*	E, K, T, R, U	Abundant in the upper montane and subalpine zones.	Presence in Uganda based on previous reports of *S. limbata* [12].	[20]
*Sticta weigelii*	-	Not found in this study.	Previous reports may represent *Sticta* sp. C.	[12,27,28,29,30,31]
*Sticta xanthotropa*	-	Not found in this study.	Previously reported as *S. weigelii* var. *xanthotropa*. The reports may represent other species with marginal flattened isidia.	[12]
*Sticta* sp. A	T*	Rare in middle and upper montane forests.	*Sticta fuliginoides* agg.	
*Sticta* sp. B	K*, T*, R	Rare in lower and middle montane forests.	*Sticta ciliata* agg. Previously reported from Rwanda as *S. ciliata* [20].	
*Sticta* sp. C	K*	Low elevation and lower montane habitats.	*Sticta weigelii* agg. May include two distinct taxa.	
*Sticta* sp. D	T*	Rare in middle and upper montane forests.		
*Sticta* sp. 2	K*, T	Rare in lower montane forests.	Presence in Tanzania based on previous reports of *S. papyracea* [12,30].	

* New record for the region.

## Data Availability

The sequence data presented in this study are openly available in the NCBI GenBank (www.ncbi.nlm.nih.gov/genbank/ accessed on 29 December 2022) with accession numbers OP999379–OP999611. The sequence alignment files and the resulting tree files from the phylogenetic analyses are openly available in the Zenodo repository (https://zenodo.org/) with doi 10.5281/zenodo.7575780 accessed on 29 December 2022.

## Supplementary Materials

The following supporting information can be downloaded at: https://www.mdpi.com/article/10.3390/jof9020246/s1, Figure S1: Bayesian tree of the genus *Sticta* based on the nuITS genetic marker region.

## Appendix A

**Table A1:** Specimen information. Specimens are collected from Kenya (Taita Hills, Mt. Kasigau, Mt. Elgon) and Tanzania (Kilimanjaro). Habitats on Mt. Kilimanjaro: Home = Chagga home garden, Cof = coffee farm, Flm = lower montane forest, FOc = middle montane *Ocotea* forest, FOD = disturbed *Ocotea* forest, FPo = upper montane *Podocarpus* forest, FPD = disturbed upper montane forest with *Erica excelsa*, FEr = subalpine *Erica trimera* forest, FED = disturbed subalpine *Erica* forest/shrubbery.

Species	Collection ID	ITS Variant	Collection Location		Accession
*Sticta afromontana*	JR10112	afromontana1	Taita Hills	Vuria	OP999402
*Sticta afromontana*	JR10121B	afromontana1	Taita Hills	Vuria	OP999404
*Sticta afromontana*	JR10189	afromontana1	Taita Hills	Yale	OP999412
*Sticta afromontana*	JR16366	afromontana1	Taita Hills	Vuria	OP999453
*Sticta afromontana*	JR25	-	Taita Hills	Yale	-
*Sticta afromontana*	JR31	-	Taita Hills	Vuria	-
*Sticta afromontana*	UK110523c	-	Taita Hills	Vuria	-
*Sticta afromontana*	UK170771f	afromontana1	Kilimanjaro	FPo1	OP999477
*Sticta afromontana*	UK170775b	afromontana3	Kilimanjaro	FPo1	OP999480
*Sticta afromontana*	UK170779c	afromontana1	Kilimanjaro	FPo1	OP999481
*Sticta afromontana*	UK170781b	afromontana1	Kilimanjaro	FPo1	OP999482
*Sticta afromontana*	UK170792d	afromontana1	Kilimanjaro	FOc2	OP999484
*Sticta afromontana*	UK170794d	-	Kilimanjaro	FOc2	-
*Sticta afromontana*	UK170800b	-	Kilimanjaro	FOc1	-
*Sticta afromontana*	UK170806j	afromontana1	Kilimanjaro	FPD4	OP999491
*Sticta afromontana*	UK170807a	-	Kilimanjaro	FPD4	-
*Sticta afromontana*	UK170817h	-	Kilimanjaro	FPD4	-
*Sticta afromontana*	UK170822k	-	Kilimanjaro	FPD4	-
*Sticta afromontana*	UK170826e	afromontana1	Kilimanjaro	FPD4	OP999496
*Sticta afromontana*	UK170830d	-	Kilimanjaro	FPD4	-
*Sticta afromontana*	UK170832c	-	Kilimanjaro	FPD4	-
*Sticta afromontana*	UK170834a	-	Kilimanjaro	FPD3	-
*Sticta afromontana*	UK170836b	-	Kilimanjaro	FPD3	-
*Sticta afromontana*	UK170841b	-	Kilimanjaro	FPD3	-
*Sticta afromontana*	UK170842e	afromontana1	Kilimanjaro	FPD3	OP999498
*Sticta afromontana*	UK170846e	afromontana1	Kilimanjaro	FPD3	OP999501
*Sticta afromontana*	UK170856c	-	Kilimanjaro	FPo4	-
*Sticta afromontana*	UK170857a	afromontana1	Kilimanjaro	FPo4	OP999503
*Sticta afromontana*	UK170858g	afromontana1	Kilimanjaro	FPo4	OP999507
*Sticta afromontana*	UK170880e	-	Kilimanjaro	FPo5	-
*Sticta afromontana*	UK170886a	afromontana1	Kilimanjaro	FPo5	OP999509
*Sticta afromontana*	UK170892b	-	Kilimanjaro	FPo5	-
*Sticta afromontana*	UK170897ar	-	Kilimanjaro	FOD4	-
*Sticta afromontana*	UK170911b	-	Kilimanjaro	FOD5	-
*Sticta afromontana*	UK170915a	-	Kilimanjaro	FOD5	-
*Sticta afromontana*	UK170930c	afromontana1	Kilimanjaro	Flm1	OP999523
*Sticta afromontana*	UK170951g	-	Kilimanjaro	Flm1	-
*Sticta afromontana*	UK171180a	afromontana4	Kilimanjaro	FOD3	OP999537
*Sticta afromontana*	UK171185h	-	Kilimanjaro	FOD3	-
*Sticta afromontana*	UK171405e	-	Kilimanjaro	FEr4	-
*Sticta afromontana*	UK171413a	-	Kilimanjaro	FEr4	-
*Sticta afromontana*	UK171438g	-	Kilimanjaro	FEr2	-
*Sticta afromontana*	UK171439t	afromontana2	Kilimanjaro	FEr2	OP999554
*Sticta afromontana*	UK171442c	-	Kilimanjaro	FEr2	-
*Sticta afromontana*	UK171445j	-	Kilimanjaro	FEr2	-
*Sticta afromontana*	UK171470v	-	Kilimanjaro	FPD1	-
*Sticta afromontana*	UK171471c	-	Kilimanjaro	FPD1	-
*Sticta afromontana*	UK171476e	afromontana1	Kilimanjaro	FPD1	OP999561
*Sticta afromontana*	UK171490e	afromontana1	Kilimanjaro	FOc3	OP999566
*Sticta afromontana*	UK171525a	-	Kilimanjaro	FPo2	-
*Sticta afromontana*	UK171526a	afromontana1	Kilimanjaro	FPo2	OP999590
*Sticta afromontana*	UK171528b	afromontana1_4	Kilimanjaro	FPo2	OP999591
*Sticta afromontana*	UK171577f	afromontana2	Kilimanjaro	FPD2	OP999595
*Sticta afromontana*	UK171578m	-	Kilimanjaro	FPD2	-
*Sticta afromontana*	UK171581j	-	Kilimanjaro	FPD2	-
*Sticta afromontana*	UK171582p	-	Kilimanjaro	FPD2	-
*Sticta afromontana*	UK171584v	afromontana1	Kilimanjaro	FPD2	OP999601
*Sticta afromontana*	UK171589b	-	Kilimanjaro	FPo3	-
*Sticta andina*	JR10117	andina1	Taita Hills	Vuria	OP999403
*Sticta aspratilis*	JR10044A	aspratilis4	Taita Hills	Shomoto Hill	OP999394
*Sticta aspratilis*	JR10057	aspratilis1	Taita Hills	Shomoto Hill	OP999395
*Sticta aspratilis*	JR10155A	aspratilis1	Taita Hills	Yale	OP999406
*Sticta aspratilis*	JR10155B	aspratilis1	Taita Hills	Yale	OP999407
*Sticta aspratilis*	JR10155C	aspratilis1	Taita Hills	Yale	OP999408
*Sticta aspratilis*	JR10171	aspratilis1	Taita Hills	Yale	OP999409
*Sticta aspratilis*	JR16103	aspratilis1	Mt. Elgon	Erica zone	OP999436
*Sticta aspratilis*	JR16107	aspratilis1	Mt. Elgon	Erica zone	OP999437
*Sticta aspratilis*	UK110551f	aspratilis2	Taita Hills	Fururu	OP999475
*Sticta aspratilis*	UK110551g	aspratilis2	Taita Hills	Fururu	OP999476
*Sticta aspratilis*	UK110566c	-	Taita Hills	Chawia	-
*Sticta aspratilis*	UK170781c	-	Kilimanjaro	FPo1	-
*Sticta aspratilis*	UK170892c	aspratilis1	Kilimanjaro	FPo5	OP999510
*Sticta aspratilis*	UK170896b	aspratilis1and2	Kilimanjaro	FPo5	OP999512
*Sticta aspratilis*	UK170910b	-	Kilimanjaro	FOD5	-
*Sticta aspratilis*	UK170912b	-	Kilimanjaro	FOD5	-
*Sticta aspratilis*	UK170916c	aspratilis1	Kilimanjaro	Flm6	OP999520
*Sticta aspratilis*	UK170975f	aspratilis1	Kilimanjaro	Cof5	OP999530
*Sticta aspratilis*	UK171478a	aspratilis1	Kilimanjaro	FOc4	OP999562
*Sticta aspratilis*	UK171487c	aspratilis1	Kilimanjaro	FOc4	OP999565
*Sticta aspratilis*	UK171508k	-	Kilimanjaro	FOD2	-
*Sticta aspratilis*	UK171515b	aspratilis3	Kilimanjaro	FOc5	OP999584
*Sticta aspratilis*	UK171562c	aspratilis1	Kilimanjaro	FED2	OP999592
*Sticta aspratilis*	UK171587j	aspratilis1	Kilimanjaro	FPo3	OP999607
*Sticta cellulosa*	UK171340t	cellulosa2	Kilimanjaro	FED1	OP999545
*Sticta cellulosa*	UK171406e	cellulosa1	Kilimanjaro	FEr4	OP999548
*Sticta cellulosa*	UK171407j	cellulosa1	Kilimanjaro	FEr4	OP999549
*Sticta cellulosa*	UK171458k	-	Kilimanjaro	FEr4	-
*Sticta cellulosa*	UK171584k	cellulosa1	Kilimanjaro	FPD2	OP999599
*Sticta ciliata*	JR16294b	ciliata1	Taita Hills	Sagalla	OP999443
*Sticta ciliata*	UK170944j	ciliata2	Kilimanjaro	Flm1	OP999528
*Sticta ciliata* (agg.)	UK110570a	-	Taita Hills	Chawia	-
*Sticta ciliata* (agg.)	UK160552r	-	Kilimanjaro	Home3	-
*Sticta ciliata* (agg.)	UK170984	-	Kilimanjaro	Cof5	-
*Sticta cyanocaperata*	UK170897f	-	Kilimanjaro	FOD4	-
*Sticta cyanocaperata*	UK170912a	cyanocaperata1	Kilimanjaro	FOD5	OP999516
*Sticta cyanocaperata*	UK170912e	cyanocaperata1	Kilimanjaro	FOD5	OP999518
*Sticta cyanocaperata*	UK171182c	cyanocaperata1	Kilimanjaro	FOD3	OP999539
*Sticta cyanocaperata*	UK171480d	cyanocaperata1	Kilimanjaro	FOc4	OP999563
*Sticta cyanocaperata*	UK171483v	-	Kilimanjaro	FOc4	-
*Sticta cyanocaperata*	UK171486b	-	Kilimanjaro	FOc4	-
*Sticta cyanocaperata*	UK171495f	cyanocaperata1	Kilimanjaro	FOc3	OP999570
*Sticta cyanocaperata*	UK171506g	-	Kilimanjaro	FOD2	-
*Sticta cyanocaperata*	UK171510m	-	Kilimanjaro	FOD2	-
*Sticta duplolimbata*	JR10194A	duplolimbata1	Taita Hills	Yale	OP999416
*Sticta duplolimbata*	JR10194B	-	Taita Hills	Yale	-
*Sticta duplolimbata*	JR16374b	duplolimbata4	Taita Hills	Vuria	OP999460
*Sticta duplolimbata*	JR16374d	duplolimbata1	Taita Hills	Vuria	OP999462
*Sticta duplolimbata*	JR68a	duplolimbata1_2_4	Taita Hills	Vuria	OP999470
*Sticta duplolimbata*	JR69	duplolimbata1	Taita Hills	Vuria	OP999471
*Sticta duplolimbata*	UK170790	duplolimbata1	Kilimanjaro	FOc2	OP999483
*Sticta duplolimbata*	UK170799d	duplolimbata1	Kilimanjaro	FOc1	OP999489
*Sticta duplolimbata*	UK170845o	-	Kilimanjaro	FPD3	-
*Sticta duplolimbata*	UK170893a	duplolimbata1	Kilimanjaro	FPo5	OP999511
*Sticta duplolimbata*	UK170914b	duplolimbata2	Kilimanjaro	FOD5	OP999519
*Sticta duplolimbata*	UK170929c	duplolimbata1	Kilimanjaro	Flm6	OP999521
*Sticta duplolimbata*	UK170931h	duplolimbata3	Kilimanjaro	Flm1	OP999524
*Sticta duplolimbata*	UK170933g	-	Kilimanjaro	Flm1	-
*Sticta duplolimbata*	UK170936g	-	Kilimanjaro	Flm1	-
*Sticta duplolimbata*	UK170950c	-	Kilimanjaro	Flm1	-
*Sticta duplolimbata*	UK170952e	duplolimbata1	Kilimanjaro	Flm1	OP999529
*Sticta duplolimbata*	UK171178f	duplolimbata1	Kilimanjaro	FOD3	OP999536
*Sticta duplolimbata*	UK171181e	duplolimbata2	Kilimanjaro	FOD3	OP999538
*Sticta duplolimbata*	UK171182b	-	Kilimanjaro	FOD3	-
*Sticta duplolimbata*	UK171188b	-	Kilimanjaro	FOD3	-
*Sticta duplolimbata*	UK171472d	-	Kilimanjaro	FPD1	-
*Sticta duplolimbata*	UK171483g	-	Kilimanjaro	FOc4	-
*Sticta duplolimbata*	UK171490f	duplolimbata1	Kilimanjaro	FOc3	OP999567
*Sticta duplolimbata*	UK171493b	-	Kilimanjaro	FOc3	-
*Sticta duplolimbata*	UK171495e	duplolimbata1	Kilimanjaro	FOc3	OP999569
*Sticta duplolimbata*	UK171497e	duplolimbata1	Kilimanjaro	FOD1	OP999572
*Sticta duplolimbata*	UK171497f	duplolimbata1	Kilimanjaro	FOD1	OP999573
*Sticta duplolimbata*	UK171502b	duplolimbata1	Kilimanjaro	FOD1	OP999574
*Sticta duplolimbata*	UK171502c	duplolimbata1	Kilimanjaro	FOD1	OP999575
*Sticta duplolimbata*	UK171503c	-	Kilimanjaro	FOD1	-
*Sticta duplolimbata*	UK171504b	duplolimbata1	Kilimanjaro	FOD2	OP999576
*Sticta duplolimbata*	UK171513f	duplolimbata1	Kilimanjaro	FOc5	OP999582
*Sticta duplolimbata*	UK171519g	duplolimbata1	Kilimanjaro	FOc5	OP999585
*Sticta duplolimbata*	UK171521h	-	Kilimanjaro	FOc5	-
*Sticta duplolimbata*	UK171586p	duplolimbata2	Kilimanjaro	FPo3	OP999605
*Sticta duplolimbata*	UK171590q	duplolimbata2	Kilimanjaro	FPo3	OP999609
*Sticta duplolimbata*	UK171596e	duplolimbata1	Kilimanjaro	Flm2	OP999610
*Sticta fuliginoides*	UK170808e	fuliginoides1	Kilimanjaro	FPD4	OP999494
*Sticta fuliginoides*	UK170844c	fuliginoides1	Kilimanjaro	FPD3	OP999499
*Sticta fuliginoides*	UK170858e	fuliginoides1	Kilimanjaro	FPo4	OP999505
*Sticta fuliginoides*	UK171438f	fuliginoides1	Kilimanjaro	FEr2	OP999552
*Sticta fuliginoides*	UK171468d	fuliginoides1	Kilimanjaro	FPD1	OP999558
*Sticta fuliginoides*	UK171485c	fuliginoides1	Kilimanjaro	FOc4	OP999564
*Sticta fuliginoides*	UK171504c	fuliginoides1	Kilimanjaro	FOD2	OP999577
*Sticta fuliginoides*	UK171577i	fuliginoides1	Kilimanjaro	FPD2	OP999597
*Sticta fuliginoides* (agg.)	UK170773c	-	Kilimanjaro	FPo1	-
*Sticta fuliginoides* (agg.)	UK170775c	-	Kilimanjaro	FPo1	-
*Sticta fuliginoides* (agg.)	UK170826g	-	Kilimanjaro	FPD4	-
*Sticta fuliginoides* (agg.)	UK170845m	-	Kilimanjaro	FPD3	-
*Sticta fuliginoides* (agg.)	UK170888g	-	Kilimanjaro	FPo5	-
*Sticta fuliginoides* (agg.)	UK170889e	-	Kilimanjaro	FPo5	-
*Sticta fuliginoides* (agg.)	UK170897g	-	Kilimanjaro	FOD4	-
*Sticta fuliginoides* (agg.)	UK171439h	-	Kilimanjaro	FEr2	-
*Sticta fuliginoides* (agg.)	UK171445h	-	Kilimanjaro	FEr2	-
*Sticta fuliginoides* (agg.)	UK171456b	-	Kilimanjaro	FEr2	-
*Sticta fuliginoides* (agg.)	UK171458m	-	Kilimanjaro	FEr4	-
*Sticta fuliginoides* (agg.)	UK171467j	-	Kilimanjaro	FEr4	-
*Sticta fuliginoides* (agg.)	UK171471d	-	Kilimanjaro	FPD1	-
*Sticta fuliginoides* (agg.)	UK171473m	-	Kilimanjaro	FPD1	-
*Sticta fuliginoides* (agg.)	UK171497ab	-	Kilimanjaro	FOD1	-
*Sticta fuliginoides* (agg.)	UK171505d	-	Kilimanjaro	FOD2	-
*Sticta fuliginoides* (agg.)	UK171514d	-	Kilimanjaro	FOc5	-
*Sticta fuliginoides* (agg.)	UK171523m	-	Kilimanjaro	FPo2	-
*Sticta fuliginoides* (agg.)	UK171582j	-	Kilimanjaro	FPD2	-
*Sticta fuliginoides* (agg.)	UK171584l	-	Kilimanjaro	FPD2	-
*Sticta fuliginoides* (agg.)	UK171586m	-	Kilimanjaro	FPo3	-
*Sticta fuliginoides* (agg.)	UK171590r	-	Kilimanjaro	FPo3	-
*Sticta fuliginosa*	JR09A46	fuliginosa1	Taita Hills	Ngangao	OP999381
*Sticta fuliginosa*	JR09D21B	fuliginosa1	Taita Hills	Ngangao	OP999384
*Sticta fuliginosa*	JR10076	fuliginosa1	Taita Hills	Vuria	OP999399
*Sticta fuliginosa*	JR10151	fuliginosa1	Taita Hills	Yale	OP999405
*Sticta fuliginosa*	JR10190B	fuliginosa1	Taita Hills	Yale	OP999413
*Sticta fuliginosa*	JR10245	fuliginosa2	Taita Hills	Mwachora	OP999425
*Sticta fuliginosa*	JR105A	fuliginosa1	Taita Hills	Vuria	OP999428
*Sticta fuliginosa*	JR105B	fuliginosa1	Taita Hills	Vuria	OP999429
*Sticta fuliginosa*	JR10K564	-	Mt. Kasigau	Plot W12	-
*Sticta fuliginosa*	JR16335	fuliginosa1	Taita Hills	Sagalla	OP999448
*Sticta fuliginosa*	JR16354	fuliginosa1	Taita Hills	Vuria	OP999450
*Sticta fuliginosa*	JR16358	fuliginosa1	Taita Hills	Vuria	OP999452
*Sticta fuliginosa*	JR16457	-	Taita Hills	Mbololo	
*Sticta fuliginosa*	JR16463	fuliginosa1	Taita Hills	Mbololo	OP999468
*Sticta fuliginosa*	JR19	-	Taita Hills	Yale	-
*Sticta fuliginosa*	JR3	fuliginosa1	Taita Hills	Yale	OP999469
*Sticta fuliginosa*	UK110504b	-	Taita Hills	Ngangao	-
*Sticta fuliginosa*	UK110512a	-	Taita Hills	Vuria	-
*Sticta fuliginosa*	UK170794g	fuliginosa1	Kilimanjaro	FOc2	OP999486
*Sticta fuliginosa*	UK170796a	fuliginosa1	Kilimanjaro	FOc2	OP999487
*Sticta fuliginosa*	UK170797j	fuliginosa1	Kilimanjaro	FOc1	OP999488
*Sticta fuliginosa*	UK170846f	fuliginosa1	Kilimanjaro	FPD3	OP999502
*Sticta fuliginosa*	UK170900h	fuliginosa1	Kilimanjaro	FOD4	OP999513
*Sticta fuliginosa*	UK170906b	fuliginosa1	Kilimanjaro	FOD4	OP999515
*Sticta fuliginosa*	UK170930b	fuliginosa1	Kilimanjaro	Flm1	OP999522
*Sticta fuliginosa*	UK171494n	fuliginosa1	Kilimanjaro	FOc3	OP999568
*Sticta fuliginosa*	UK171504m	fuliginosa1	Kilimanjaro	FOD2	OP999579
*Sticta fuliginosa*	UK171594e	-	Kilimanjaro	Flm2	-
*Sticta marginalis*	JR_58_7	marginalis3	Mt. Kasigau	Plot W14	OP999379
*Sticta marginalis*	JR090X8a	-	Taita Hills	Chawia	-
*Sticta marginalis*	JR09D12A	marginalis1	Taita Hills	Ngangao	OP999383
*Sticta marginalis*	JR10028B	marginalis1	Taita Hills	Vuria	OP999391
*Sticta marginalis*	JR10176A	marginalis1	Taita Hills	Yale	OP999410
*Sticta marginalis*	JR10193A	marginalis1	Taita Hills	Yale	OP999415
*Sticta marginalis*	JR10202B	marginalis1	Taita Hills	Yale	OP999417
*Sticta marginalis*	JR10K532	-	Mt. Kasigau	Plot W9	-
*Sticta marginalis*	JR10K552A	-	Mt. Kasigau	Plot W11	-
*Sticta marginalis*	JR10K552B	-	Mt. Kasigau	Plot W11	-
*Sticta marginalis*	JR10K563	-	Mt. Kasigau	Plot W12	-
*Sticta marginalis*	JR110030A	marginalis4	Taita Hills	Vuria	OP999432
*Sticta marginalis*	JR110055	marginalis1	Taita Hills	Vuria	OP999433
*Sticta marginalis*	JR16248	marginalis1	Taita Hills	Mbololo	OP999441
*Sticta marginalis*	JR16294a	-	Taita Hills	Sagalla	-
*Sticta marginalis*	JR16312	marginalis1	Taita Hills	Sagalla	OP999446
*Sticta marginalis*	JR16322a	marginalis1	Taita Hills	Sagalla	OP999447
*Sticta marginalis*	JR16420	-	Taita Hills	Mbololo	-
*Sticta marginalis*	JR16440	-	Taita Hills	Mbololo	-
*Sticta marginalis*	UK110501a	-	Taita Hills	Ngangao	-
*Sticta marginalis*	UK110535b	-	Taita Hills	Vuria	-
*Sticta marginalis*	UK170800m	marginalis2	Kilimanjaro	FOc1	OP999490
*Sticta marginalis*	UK170927a	-	Kilimanjaro	Flm6	-
*Sticta marginalis*	UK170932b	marginalis1	Kilimanjaro	Flm1	OP999525
*Sticta marginalis*	UK170933h	marginalis1	Kilimanjaro	Flm1	OP999526
*Sticta marginalis*	UK170934b	-	Kilimanjaro	Flm1	-
*Sticta marginalis*	UK170937j	-	Kilimanjaro	Flm1	-
*Sticta marginalis*	UK170942c	marginalis1	Kilimanjaro	Flm1	OP999527
*Sticta marginalis*	UK170943d	-	Kilimanjaro	Flm1	-
*Sticta marginalis*	UK170950d	-	Kilimanjaro	Flm1	-
*Sticta marginalis*	UK171183a	marginalis1	Kilimanjaro	FOD3	OP999540
*Sticta marginalis*	UK171183b	-	Kilimanjaro	FOD3	-
*Sticta marginalis*	UK171186a	-	Kilimanjaro	FOD3	-
*Sticta marginalis*	UK171504k	marginalis1	Kilimanjaro	FOD2	OP999578
*Sticta marginalis*	UK171596k	marginalis1	Kilimanjaro	Flm2	OP999611
*Sticta munda*	UK170888c	-	Kilimanjaro	FPo5	-
*Sticta munda*	UK171497d	munda3	Kilimanjaro	FOD1	OP999571
*Sticta munda*	UK171508j	munda1	Kilimanjaro	FOD2	OP999580
*Sticta munda*	UK171510k	munda1	Kilimanjaro	FOD2	OP999581
*Sticta munda*	UK171582i	munda2	Kilimanjaro	FPD2	OP999598
*Sticta munda*	UK171584u	munda1	Kilimanjaro	FPD2	OP999600
*Sticta munda*	UK171586k	munda1	Kilimanjaro	FPo3	OP999604
*Sticta munda*	UK171586l	-	Kilimanjaro	FPo3	-
*Sticta sublimbata*	JR09A60	sublimbata1	Taita Hills	Ngangao	OP999382
*Sticta sublimbata*	JR09D6A	sublimbata1	Taita Hills	Ngangao	OP999385
*Sticta sublimbata*	JR09W12a	sublimbata8	Mt. Kasigau	-	OP999387
*Sticta sublimbata*	JR09Y12a	sublimbata6	Taita Hills	Mbololo	OP999388
*Sticta sublimbata*	JR09Y15b	sublimbata6	Taita Hills	Mbololo	OP999389
*Sticta sublimbata*	JR09Z2	sublimbata1	Taita Hills	Ngangao	OP999390
*Sticta sublimbata*	JR10032A	sublimbata2	Taita Hills	Shomoto Hill	OP999392
*Sticta sublimbata*	JR10039B	sublimbata2	Taita Hills	Shomoto Hill	OP999393
*Sticta sublimbata*	JR10066A	sublimbata2	Taita Hills	Shomoto Hill	OP999397
*Sticta sublimbata*	JR10066B	sublimbata2	Taita Hills	Shomoto Hill	OP999398
*Sticta sublimbata*	JR10191	sublimbata1	Taita Hills	Yale	OP999414
*Sticta sublimbata*	JR10208A	sublimbata5	Taita Hills	Yale	OP999418
*Sticta sublimbata*	JR10214B	sublimbata1	Taita Hills	Yale	OP999420
*Sticta sublimbata*	JR10220A	sublimbata2	Taita Hills	Mwachora	OP999421
*Sticta sublimbata*	JR10228D	sublimbata1	Taita Hills	Mwachora	OP999422
*Sticta sublimbata*	JR10230A	sublimbata1	Taita Hills	Mwachora	OP999423
*Sticta sublimbata*	JR10241	sublimbata1	Taita Hills	Mwachora	OP999424
*Sticta sublimbata*	JR10282B	sublimbata1	Taita Hills	Macha	OP999427
*Sticta sublimbata*	JR10K494	-	Mt. Kasigau	Plot W5	-
*Sticta sublimbata*	JR10K495	-	Mt. Kasigau	Plot W5	-
*Sticta sublimbata*	JR16275	sublimbata9	Taita Hills	Sagalla	OP999442
*Sticta sublimbata*	JR16355	sublimbata2	Taita Hills	Vuria	OP999451
*Sticta sublimbata*	JR16367a	sublimbata2	Taita Hills	Vuria	OP999454
*Sticta sublimbata*	JR16367b	sublimbata2	Taita Hills	Vuria	OP999455
*Sticta sublimbata*	JR16373	sublimbata4	Taita Hills	Vuria	OP999458
*Sticta sublimbata*	JR16374a	sublimbata1	Taita Hills	Vuria	OP999459
*Sticta sublimbata*	JR16374c	sublimbata2	Taita Hills	Vuria	OP999461
*Sticta sublimbata*	JR16417	sublimbata3	Taita Hills	Mbololo	OP999463
*Sticta sublimbata*	JR16419	sublimbata7	Taita Hills	Mbololo	OP999464
*Sticta sublimbata*	JR16443	sublimbata8	Taita Hills	Mbololo	OP999466
*Sticta sublimbata*	UK110502	-	Taita Hills	Ngangao	-
*Sticta sublimbata*	UK110510a	-	Taita Hills	Vuria	-
*Sticta sublimbata*	UK110520a	-	Taita Hills	Vuria	-
*Sticta sublimbata*	UK110539d	-	Taita Hills	Vuria	-
*Sticta sublimbata*	UK110547a	sublimbata1	Taita Hills	Fururu	OP999473
*Sticta sublimbata*	UK110547b	sublimbata1	Taita Hills	Fururu	OP999474
*Sticta sublimbata*	UK110554a	-	Taita Hills	Chawia	-
*Sticta sublimbata*	UK110556a	-	Taita Hills	Chawia	-
*Sticta sublimbata*	UK160482e	-	Kilimanjaro	Home1	-
*Sticta sublimbata*	UK160552m	-	Kilimanjaro	Home3	-
*Sticta sublimbata*	UK160557f	-	Kilimanjaro	Home3	-
*Sticta sublimbata*	UK160559b	-	Kilimanjaro	Home3	-
*Sticta tomentosa*	JR10086	tomentosa1	Taita Hills	Vuria	OP999400
*Sticta tomentosa*	JR10095A	tomentosa1	Taita Hills	Vuria	OP999401
*Sticta tomentosa*	JR10260	tomentosa1	Taita Hills	Mwachora	OP999426
*Sticta tomentosa*	JR16352	tomentosa1	Taita Hills	Vuria	OP999449
*Sticta tomentosa*	JR16367c	tomentosa1	Taita Hills	Vuria	OP999456
*Sticta tomentosa*	JR16368	tomentosa1	Taita Hills	Vuria	OP999457
*Sticta tomentosa*	UK110523b	tomentosa1	Taita Hills	Vuria	OP999472
*Sticta tomentosa*	UK170912c	tomentosa1	Kilimanjaro	FOD5	OP999517
*Sticta tomentosa*	UK170934c	-	Kilimanjaro	Flm1	-
*Sticta tomentosa*	UK170936f	-	Kilimanjaro	Flm1	-
*Sticta tomentosa*	UK170943c	-	Kilimanjaro	Flm1	-
*Sticta tomentosa*	UK170950b	-	Kilimanjaro	Flm1	-
*Sticta tomentosa*	UK171473j	-	Kilimanjaro	FPD1	-
*Sticta umbilicariiformis*	JR16098	umbilicariiformis1	Mt. Elgon	Erica zone	OP999434
*Sticta umbilicariiformis*	JR16102	umbilicariiformis3	Mt. Elgon	Erica zone	OP999435
*Sticta umbilicariiformis*	JR16108	umbilicariiformis1	Mt. Elgon	Erica zone	OP999438
*Sticta umbilicariiformis*	UK170774k	umbilicariiformis3	Kilimanjaro	FPo1	OP999478
*Sticta umbilicariiformis*	UK170775a	umbilicariiformis3	Kilimanjaro	FPo1	OP999479
*Sticta umbilicariiformis*	UK170794c	-	Kilimanjaro	FOc2	-
*Sticta umbilicariiformis*	UK170806m	umbilicariiformis3	Kilimanjaro	FPD4	OP999492
*Sticta umbilicariiformis*	UK170808c	umbilicariiformis3	Kilimanjaro	FPD4	OP999493
*Sticta umbilicariiformis*	UK170821d	-	Kilimanjaro	FPD4	-
*Sticta umbilicariiformis*	UK170821h	umbilicariiformis1	Kilimanjaro	FPD4	OP999495
*Sticta umbilicariiformis*	UK170826f	-	Kilimanjaro	FPD4	-
*Sticta umbilicariiformis*	UK170842d	umbilicariiformis3	Kilimanjaro	FPD3	OP999497
*Sticta umbilicariiformis*	UK170846d	umbilicariiformis3	Kilimanjaro	FPD3	OP999500
*Sticta umbilicariiformis*	UK170858d	umbilicariiformis3	Kilimanjaro	FPo4	OP999504
*Sticta umbilicariiformis*	UK171102b	umbilicariiformis1	Kilimanjaro	FED5	OP999531
*Sticta umbilicariiformis*	UK171106b	umbilicariiformis2	Kilimanjaro	FED5	OP999532
*Sticta umbilicariiformis*	UK171119b	umbilicariiformis5	Kilimanjaro	FED5	OP999533
*Sticta umbilicariiformis*	UK171131a	umbilicariiformis5	Kilimanjaro	FED4	OP999534
*Sticta umbilicariiformis*	UK171131b	umbilicariiformis1	Kilimanjaro	FED4	OP999535
*Sticta umbilicariiformis*	UK171320e	umbilicariiformis4	Kilimanjaro	FED1	OP999541
*Sticta umbilicariiformis*	UK171320f	umbilicariiformis4	Kilimanjaro	FED1	OP999542
*Sticta umbilicariiformis*	UK171320g	umbilicariiformis4	Kilimanjaro	FED1	OP999543
*Sticta umbilicariiformis*	UK171337	umbilicariiformis1	Kilimanjaro	FED1	OP999544
*Sticta umbilicariiformis*	UK171340s	-	Kilimanjaro	FED1	-
*Sticta umbilicariiformis*	UK171404e	umbilicariiformis1	Kilimanjaro	FEr4	OP999546
*Sticta umbilicariiformis*	UK171405f	umbilicariiformis1	Kilimanjaro	FEr4	OP999547
*Sticta umbilicariiformis*	UK171411c	umbilicariiformis4	Kilimanjaro	FEr4	OP999550
*Sticta umbilicariiformis*	UK171429	umbilicariiformis4	Kilimanjaro	FEr3	OP999551
*Sticta umbilicariiformis*	UK171433a	-	Kilimanjaro	FEr3	-
*Sticta umbilicariiformis*	UK171435f	-	Kilimanjaro	FEr3	-
*Sticta umbilicariiformis*	UK171438h	umbilicariiformis4	Kilimanjaro	FEr2	OP999553
*Sticta umbilicariiformis*	UK171439u	umbilicariiformis1	Kilimanjaro	FEr2	OP999555
*Sticta umbilicariiformis*	UK171442b	-	Kilimanjaro	FEr2	-
*Sticta umbilicariiformis*	UK171449i	umbilicariiformis4	Kilimanjaro	FEr2	OP999556
*Sticta umbilicariiformis*	UK171455h	umbilicariiformis1	Kilimanjaro	FEr2	OP999557
*Sticta umbilicariiformis*	UK171458h	-	Kilimanjaro	FEr4	-
*Sticta umbilicariiformis*	UK171467k	-	Kilimanjaro	FEr4	-
*Sticta umbilicariiformis*	UK171468e	umbilicariiformis3	Kilimanjaro	FPD1	OP999559
*Sticta umbilicariiformis*	UK171468f	umbilicariiformis3	Kilimanjaro	FPD1	OP999560
*Sticta umbilicariiformis*	UK171494k	-	Kilimanjaro	FOc3	-
*Sticta umbilicariiformis*	UK171514c	umbilicariiformis3	Kilimanjaro	FOc5	OP999583
*Sticta umbilicariiformis*	UK171521f	umbilicariiformis3	Kilimanjaro	FOc5	OP999588
*Sticta umbilicariiformis*	UK171524b	umbilicariiformis3	Kilimanjaro	FPo2	OP999589
*Sticta umbilicariiformis*	UK171563a	umbilicariiformis2	Kilimanjaro	FED2	OP999593
*Sticta umbilicariiformis*	UK171569d	umbilicariiformis4	Kilimanjaro	FED2	OP999594
*Sticta umbilicariiformis*	UK171577h	umbilicariiformis1	Kilimanjaro	FPD2	OP999596
*Sticta umbilicariiformis*	UK171578t	-	Kilimanjaro	FPD2	-
*Sticta umbilicariiformis*	UK171584x	umbilicariiformis2	Kilimanjaro	FPD2	OP999602
*Sticta umbilicariiformis*	UK171584y	umbilicariiformis1	Kilimanjaro	FPD2	OP999603
*Sticta umbilicariiformis*	UK171590j	umbilicariiformis3	Kilimanjaro	FPo3	OP999608
*Sticta* sp. A (*fuliginoides* agg.)	UK170858f	A1	Kilimanjaro	FPo4	OP999506
*Sticta* sp. A (*fuliginoides* agg.)	UK170884b	A1	Kilimanjaro	FPo5	OP999508
*Sticta* sp. A (*fuliginoides* agg.)	UK171519h	A1	Kilimanjaro	FOc5	OP999586
*Sticta* sp. A (*fuliginoides* agg.)	UK171521g	-	Kilimanjaro	FOc5	-
*Sticta* sp. B (*ciliata* agg.)	JR09D8A	B1	Taita Hills	Ngangao	OP999386
*Sticta* sp. B (*ciliata* agg.)	JR10060C	B2	Taita Hills	Shomoto Hill	OP999396
*Sticta* sp. B (*ciliata* agg.)	UK170792q	B1	Kilimanjaro	FOc2	OP999485
*Sticta* sp. B (*ciliata* agg.)	UK170901b	B1	Kilimanjaro	FOD4	OP999514
*Sticta* sp. C (*weigelii* agg.)	JR090X3	C1	Taita Hills	Chawia	OP999380
*Sticta* sp. C (*weigelii* agg.)	JR10180B	C1	Taita Hills	Yale	OP999411
*Sticta* sp. C (*weigelii* agg.)	JR10180C	-	Taita Hills	Yale	-
*Sticta* sp. C (*weigelii* agg.)	JR10212B	C1	Taita Hills	Yale	OP999419
*Sticta* sp. C (*weigelii* agg.)	JR14634/082	-	Taita Hills	Maktau Hill	-
*Sticta* sp. C (*weigelii* agg.)	JR16201a	C2	Taita Hills	Fururu	OP999439
*Sticta* sp. C (*weigelii* agg.)	JR16201b	C2	Taita Hills	Fururu	OP999440
*Sticta* sp. C (*weigelii* agg.)	JR16295	C1	Taita Hills	Sagalla	OP999444
*Sticta* sp. C (*weigelii* agg.)	JR16310	C1	Taita Hills	Sagalla	OP999445
*Sticta* sp. C (*weigelii* agg.)	JR16439	C1	Taita Hills	Mbololo	OP999465
*Sticta* sp. C (*weigelii* agg.)	JR16462	C1	Taita Hills	Mbololo	OP999467
*Sticta* sp. C (*weigelii* agg.)	UK110555e	-	Taita Hills	Chawia	-
*Sticta* sp. D	UK171519k	D1	Kilimanjaro	FOc5	OP999587
*Sticta* sp. D	UK171586q	D1	Kilimanjaro	FPo3	OP999606
*Sticta* sp. 2	JR09Y12b	-	Taita Hills	Mbololo	-
*Sticta* sp. 2	JR09Y13	-	Taita Hills	Mbololo	-
*Sticta* sp. 2	JR10K302	sp. 2	Mt. Kasigau	Plot E15	OP999430
*Sticta* sp. 2	JR10K303	sp. 2	Mt. Kasigau	Plot E15	OP999431
