# Supplementary material for: The Lichen Genus Sticta (Lobariaceae, Peltigerales) in East African Montane Ecosystems
Source: J Fungi (Basel). 2023 Feb 12;9(2):246. doi: 10.3390/jof9020246 (PMC9961217; doi:10.3390/jof9020246)

Figure S1. Bayesian tree of the genus *Sticta* based on the nuITS genetic marker region. Newly obtained sequences are in black, number after the species name refers to ITS variant (specimens and ITS variants are listed in Table A1). The colored polygons (rectangle, triangle) show the distribution of the specimens with the ITS variant. On Mt. Kilimanjaro (rectangles), each vegetation zone is indicated with color (see label) and disturbed habitats are further separated with grid; the width of the rectangle indicates the number of sample plots in which the specimens were present. In the Taita Hills, each triangle indicates presence in one forest fragment. On Mt. Kasigau, each triangle refers to presence on one collection transect, corresponding to the northern, eastern, southern, and western slopes of the mountain. The posterior probability values (PP) are presented on the branches, thicker branch indicating higher support value (see label). The scale refers to nucleotide substitutions per site. A–F refer to portions of the tree shown in the figures within the publication.

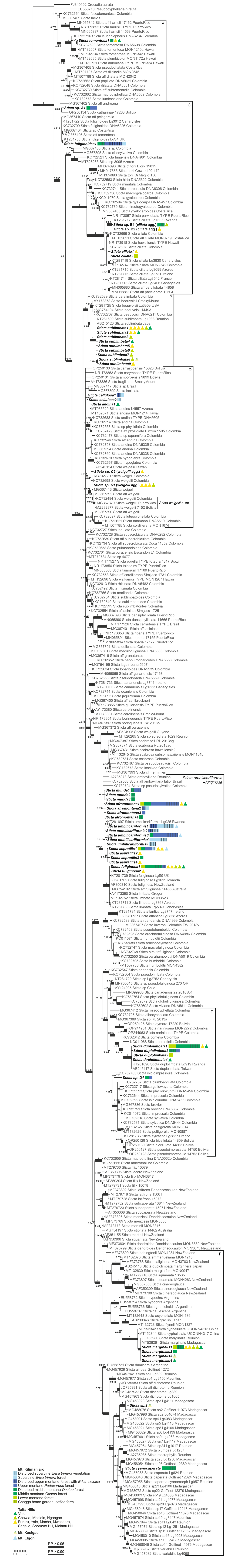

Supplement: Supplementary file 1 [file jof-09-00246-s001.zip › jof-2164798-supplementary.pdf]
